# Phylogeography of the striped field mouse (*Apodemus agrarius* Pallas, 1771) in light of new data from central part of Northern Eurasia

**DOI:** 10.1371/journal.pone.0276466

**Published:** 2022-10-20

**Authors:** Lidia Yalkovskaya, Petr Sibiryakov, Aleksandr Borodin

**Affiliations:** Institute of Plant and Animal Ecology, Ural Branch of the Russian Academy of Sciences, Yekaterinburg, Russia; University of Helsinki: Helsingin Yliopisto, FINLAND

## Abstract

A phylogeographic analysis of *A*. *agrarius* based on the complete mtDNA cytochrome *b* and control region sequences has been performed using data obtained for the first time for the species from large regions of the central part of Northern Eurasia (23 localities of Altai, Western Siberia, and the Urals). The obtained results have demonstrated a complex intraspecific differentiation of *A*. *agrarius*, which has manifested not only in the isolation of the isles populations in Southeast Asia (Jeju and Taiwan), but also in the genetic heterogeneity of mainland populations, which has reflected the history of the modern intraspecific genetic diversity formation against the background of changing physiographic conditions of Eurasia in the Quaternary. The divergence of genetic lineages has taken place apparently simultaneously (in mid-Pleistocene) on the territory of the Eastern part of the modern disjunctive range, where all the identified lineages are present today. The demographic history and possible evolutionary scenarios for *A*. *agrarius* in the Western part of the range have been considered. TMRC reconstructions have shown that the lifetime of the common ancestor of the lineage that expanded in the Western Palearctic is about 17.7 [95% HPD 13.2–22.5] kyr. This suggests that the transcontinental expansion of *A*. *agrarius* is a relatively recent event that has occurred after the LGM.

## Introduction

The study of genetic differentiation and phylogeography of widespread species is important while researching modern biodiversity, formation of species ranges, effects of global climate change and human impact, and biological expansion and invasion problems. Striped field mouse (*Apodemus agrarius* Pallas, 1771), the most widespread and abundant species of the genus *Apodemus*, inhabits forest and forest-steppe zones, and occupies almost the entire temperate zone of the Palearctic from the Korean Peninsula and the islands of the East China Sea to the territories of Northern and Western Europe [1, 2]. The modern range of *A*. *agrarius* is divided in two geographically well-isolated parts: the Western part (Europe–Siberia–Kazakhstan) and the Eastern part (Far East–China). The disjunction takes place in the arid and mountainous regions of Transbaikalia and Mongolia, where there are no suitable conditions for the species’ existence at present. An expansion of the boundaries of the species range has been observed in both its parts in recent decades, and is associated with both climate change and human activity [1, 3–6].

By now, a considerable amount of data on the genetic differentiation, phylogeography, and evolutionary history of *A*. *agrarius* has been obtained by analyzing the mitochondrial genome. The phylogenetic relations of continental and island populations of South Korea [7–9], the genetic diversity of populations of the Russian Far East and isolated populations in the Far North (Magadan Region) [10, 11], and the history of colonization of the Danish islands by striped field mouse [12] have all been studied this way. Hypotheses regarding the time, consecutiveness and the possible causes of intraspecific genetic differentiation and the history of the formation of the modern *A*. *agrarius* range have been proposed based on cytochrome *b* gene data [10, 11, 13–17], which have been confirmed in a recent work involving significant amount of material from both parts of the species distribution area [18]. However, the question of the possible *A*. *agrarius* differentiation inside the mainland part of the range still remains open. The studies using the complete cytochrome *b* sequences have not focused on this problem [9, 11, 17], but the analysis of partial cytochrome *b* sequences has demonstrated contradictory results. Some studies have suggested the existence of several mainland lineages of *A*. *agrarius* [16]; the others have shown the genetic homogeneity of mainland populations [18]. The question of the phylogenetic relationships between the modern populations of *A*. *agrarius* from Western and Eastern Palearctic also remains unclear. In addition, despite the significant geographical coverage of the studies, the central part of Northern Eurasia has been practically unstudied, i.e. the phylogeographic analysis has not accounted for data from a large region located on the path of transcontinental expansion of *A*. *agrarius* from the East to the Western Palearctic and characterized by peculiar geological history and physiographic conditions.

This study presents the results of mtDNA variability analysis of the striped field mouse using data from the Altai, Western Siberia and Urals populations, which previously have not been explored. It aims to conduct the phylogenetic and phylogeographic reconstructions using mtDNA cytochrome *b* and control region data while taking into account the new data obtained from the central part of Northern Eurasia with particular attention paid to the genetic structure of mainland populations and to the history of Western Palearctic colonization by the species.

## Materials and methods

### Samples and sequence data

**Fig 1:**
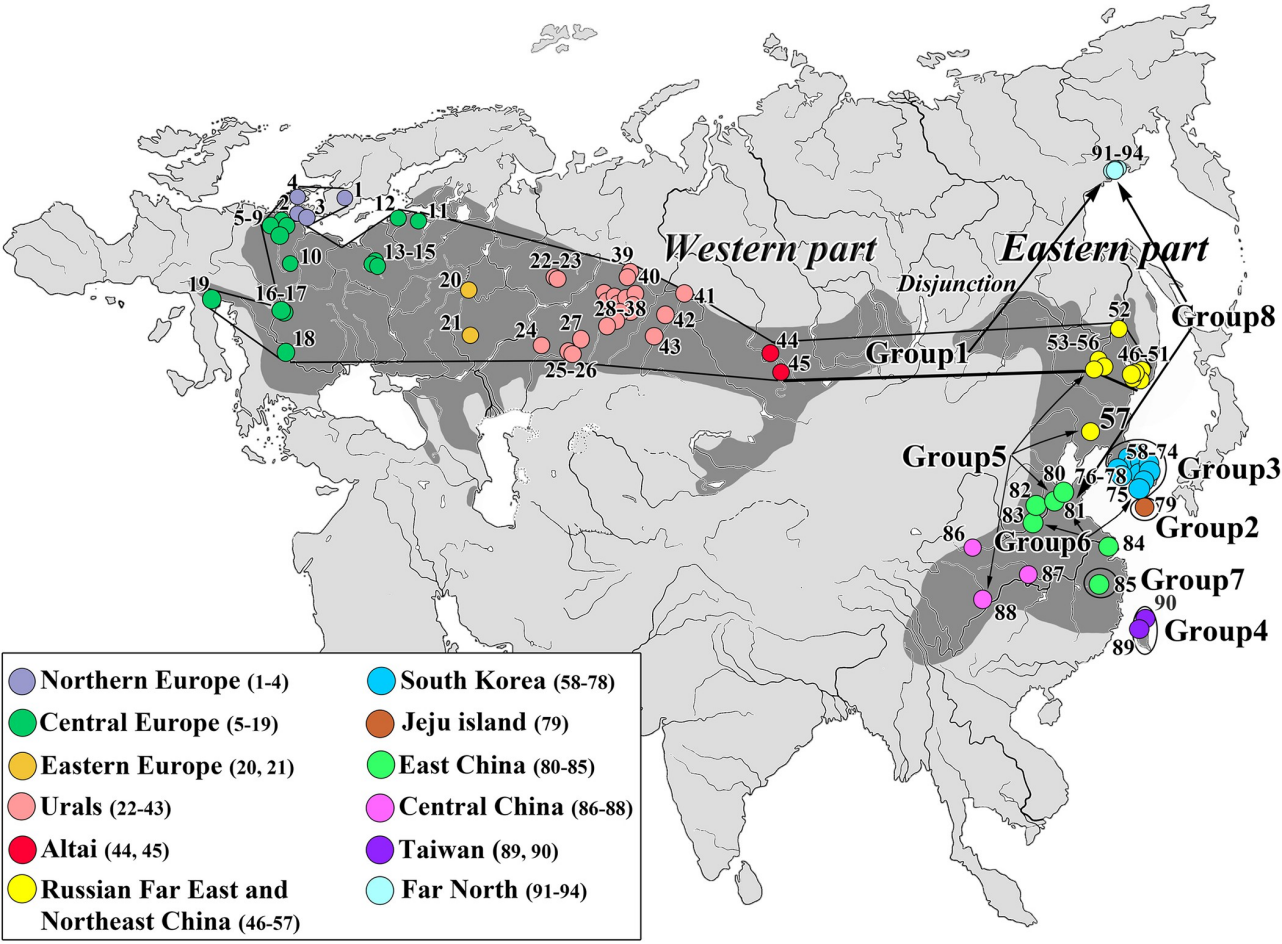
*A*. *agrarius* range (dark grey area) and localization of samples used in the study. The numbers of localities correspond to those given in Table 1.

The muscle tissue of 138 striped field mice (*Apodemus agrarius* Pallas, 1771) from 23 localities of central part of the North Eurasia (Cis–Urals, Southern, Middle and Northern Urals, Western Siberia and Altai) has been used (Table 1, Fig 1, S1 Table). The study has been carried out using personal collections and specimens from the collection of the Museum of the Institute of Plant and Animal Ecology Ural Branch Russian Academy of Sciences, Yekaterinburg. All work has been carried out in accordance with the European Convention for the protection of vertebrate animals used for experiments or other scientific purposes.

**Table 1:** *A*. *agrarius* samples analyzed in the study.

Region	Locality	Map reference		Number of sequences/haplotypes				
				*cyt b*		CR		*cyt b* + CR
Original data								
Cis-Urals	Belaya Kholunitsa	22			2/1	2/2	2/2	
	Chernaya Kholunitsa	23			2/1	2/2	2/2	
	Kungur	28			1	2/2	1	
	Talovskaya Steppe	24			5/4	5/3	5/4	
Southern Urals	Aytuarskaya Steppe	25			5/2	5/2	5/2	
	Mt. Verblyzhka	26			1	1	1	
	Kinzebulatovo	27			2/2	1	1	
	Baturinsky	29			5/3	5/4	5/4	
	Il’meny	31			2/2	2/2	2/2	
	East Ural Reserve	30, 32			15/8	22/11	13/8	
Middle Urals	Starikovo	33			6/4	6/4	6/5	
	Dvurechensk	34			5/3	5/3	5/3	
	Yekaterinburg	35			3/2	4/2	2/2	
	Shigaevo	36			4/3	6/4	4/3	
	Khomutovka	38			6/2	8/3	6/2	
	Kharlovskoe	37			7/5	7/6	7/6	
	Nizhny Tagil	40			24/8	24/13	24/13	
Northern Urals	Sos’va	39			7/2	7/4	7/4	
Western Siberia	Tomilovo	41			3/3	2/2	2/2	
	Mashkara	42			4/3	4/4	4/4	
	Zverinogolovskoe	43			4/2	4/2	4/2	
Altai	Bol’shaya Rechka	44			2/2	2/2	2/2	
	Surtayka	45			7/3	7/3	7/3	
GenBank data§								
Northern Europe	Sweden	1	1			1	1	
	Denmark	2–4	50/6			53/10	50/14	
Central Europe	Estonia	11, 12	3/3			3/2	3/3	
	Germany	5–9	13/3			17/10	12/12	
	Czech Republic	10	–			1	–	
	Poland	13–15	12/6			12/10	12/10	
	Hungary	16, 17	7/2			–	–	
	Serbia	18	1			–	–	
	Italy	19	1			–	–	
Eastern Europe	Russia	20, 21	3/3			1	–	
Russian Far East		46–51	35/9			–	–	
North-Eastern China		52–57	12/11			3/3	3/3	
Eastern China		80–85	26/14			–	–	
Central China		86–88	4/4			10/10	–	
South Korea		58–74	36/28			2/2	2/2	
South Korea small islands		75–78	16/10			–	–	
Jeju Island		79	65/31			1	1	
Taiwan		89, 90	17/8			–	–	
Far North isolate	Magadan Region	91–94	80/5			–	–	

§ [9, 11–15, 17, 19–26]

Complete cytochrome *b* gene sequences (*cyt b*– 1140 bp) for 122 specimens, complete mtDNA control region sequences (CR– 864 bp) for 133 specimens and concatenated sequences of the two mitochondrial markers (2004 bp) for 117 specimens have been obtained (Table 1).

The analysis of the *cyt b* variable sites distribution has been carried out using 593 complete and partial sequences, including both original and GenBank data (S1 and S2 Tables).

In the phylogeographic analysis the following published data has been used: *cyt b*– 382 complete sequences and 153 haplotypes; CR– 104 complete sequences and 48 haplotypes; 84 concatenated sequences and 45 haplotypes (Table 1, S1 Table). *Apodemus chevrieri* (GenBank: HQ896683), *Apodemus draco* (GenBank: HQ333255), *Apodemus latronum* (GenBank: HQ333256) and *Apodemus peninsulae* (GenBank: HQ660074, JN546584, KP671850) have been used as an outgroup.

### DNA extraction, amplification, and sequencing

Total genomic DNA has been extracted from small pieces of muscle tissue preserved in 95% ethanol using the method of salt extraction [27].

PCR of the mtDNA fragments containing the *cyt b* has been performed in two variants. First, one fragment of the mtDNA about 1200 bp length containing full *cyt b* gene has been amplified using a pair of primers: L7 (5’-ACCAATGACATGAAAAATCATCGTT-3’) and H6 (5’-TCTCCATTTCTGGTTTACAAGAC-3’) [28]. PCR has been carried out in 25 μL of reaction mixture (3 mM of each of the dNTPs, 1x *Taq* Buffer (+KCl), 2.5 mM MgCl_2_, 7.5 pM of each primer, 2.5U *Taq* DNA Polymerase, and 50–100 ng DNA template) according to the protocol (95°C for 5 min; [95°C for 20 s, 58°C for 15 s, 72°C for 1 min 20 s], 35 cycles; 72°C for 10 min). In case of lack of results from the first PCR variant, two more primers–H2 (5′-TAGTTGTCTGGGTCTCC-3′) and L8 (5′-CTGCCATGAGGACAAATATCATT-3′) [29]–have been used. Two fragments of the *cyt b* about 550 bp and 700 bp length have been amplified using two pairs of primers: L7 –H2, and L8 –H6, respectively. The amplification reaction has been carried out in 25 μL of the same reaction mixture as in the first PCR variant according to the protocol (95°C for 5 min; [95°C for 20 s, 55°C for 15 s, 72°C for 45 s], 30 cycles; 72°C for 5 min).

The mtDNA fragments containing the CR have been amplified in two segments using two pairs of primers: 1 (5’-ATAAACATTACTCGGTCTTGTAAC-3’)– 2bis (5’-CACAGTTATGGAAGTCTTGG-3’) and 3 (5’-CGTTCCCCTAAATAAGAC-3’)– 4 (5’-TAATTATAAGGCCAGGACCA-3’) [20]. PCR has been carried out in 50 μL of reaction mixture (10 mM of each of the dNTPs, 1x *Taq* Buffer (+KCl), 2.5 mM MgCl_2_, 7.5 pM of each primer, 2.5U *Taq* DNA Polymerase, and 50–100 ng DNA template) according to the protocol (95°C for 5 min; [95°C for 30 s, 55°C for 1 min, 72°C for 1 min], 31 cycles; 72°C for 15 min).

The PCR products have been purified and directly sequenced using either the Big Dye Terminator Cycle Sequencing Kit V.3.1 (Applied Biosystems, USA) or Bright Dye Terminator Cycle Sequencing Kit (NimaGen, Netherlands) in two directions with the same primers as in PCR, followed by sequence determination on an ABI Prism 3130 automated analyzer (Applied Biosystems, USA).

### Phylogenetic, phylogeographical and genetic structure analysis

The chromatograms have been analyzed using the BioEdit v7.2.0 (4.30.2013) software program [30]. The sequence alignment, calculation of genetic distances, and construction of phylogenetic trees with the neighbor joining (NJ) and maximum likelihood (ML) methods have been carried out in the MEGA v6 software program [31] using the Bayesian inference (BI) as implemented in the MrBayes v3.2.2 software program [32]. The search for optimum models of nucleotide sequence evolution has been performed in the MrModeltest 2.3 software program [33]. The construction of median-joining networks (MJ network) has been carried out in the Network v5.0.0.0 software program [34]. The assessment of genetic diversity indexes and the tests for selective neutrality have been performed in the Arlequin v 3.1 [35] and DnaSP v.5.10 [36] software programs. The marker congruence has been evaluated using the partition homogeneity test (HOMPART) [37] in the PAUP v. 4.0*b*10 software package [38]. To analyze genetic distances, the Maximum Composite Likelihood model has been used.

In order to construct the trees by the ML method, the Hasegawa-Kishino-Yano model (HKY) with the proportion of invariant sites (+ I) and normalization by gamma distribution (+ G) has been chosen. When constructing phylogenetic trees using the NJ method to calculate distances, the Maximum Composite Likelihood model has been used. When the phylogenetic tree on the basis of the *cyt b* and CR sequences has been reconstructed using the BI method, a complex approach with a separate choice of a model for each of the three codon positions has been used. For the first and second codon position, it has been Kimura’s model (K81) +G; and for the third position, HKY+G. When the phylogenetic tree has been reconstructed using the BI on the basis of the concatenated sequences, a complex approach with a separate choice of a model for each of the three positions in the *cyt b* codon and for the CR has been used. For the first and second codon positions it has been K81+G; for the third position, HKY; and for the CR, HKY+G. In order to construct the phylogenetic trees using the ML and NJ methods, the statistical testing of the tree topology has been carried out using the bootstrap analysis (1000 cycles). In the case of used BI for phylogenetic trees, two parallel analyses consisting of four Markov chains, each for 10 000 000 cycles, have been run simultaneously, with sampling every 500th cycle and removing the first 5001 cycles as the burn-in stage.

For time to most recent common ancestor (tMRCA) reconstruction for *A*. *agrarius* of Group 1 based on concatenated sequences, the BEAST 2.3.1 package [39] has been used. The following accumulation rates of substitutions have been regarded: 1.863–3.974×10^−7^ sub per site y^−1^ for *cyt b* and 1.975–4.208×10^−7^ for CR sub per site y^−1^ [12]. The analysis has been carried out using the HKY substitution model and strict molecular clock. Each 1000th step from 100000000 generations discarding the first 25% as burn-in has been collected. For model selection, path sampling and stepping-stone sampling have been used. HKY, GTR and JC69 substitution models, and strict, lognormal and random local molecular clock models have been compared.

## Results

### Description of data

The complete mitochondrial *cyt b* gene (1140 bp) for 122 *A*. *agrarius* specimens from 23 localities from the central part of the North Eurasia (Cis-Urals, Southern, Middle and Northern Urals, Western Siberia and Altai) has been sequenced, and 55 variable sites (22 of which have been informative for parsimony analysis), 49 transitions, and 6 transversions have been revealed. C+G composition has been about 40%. Of 122 *cyt b* sequences, 41 haplotypes have been observed (Table 1, S1 Table), 39 of which were new (GenBank № OM970127–OM970165), and two haplotypes detected in Western Siberia, Southern and Middle Urals have been previously found in the central part of European Russia [17]. The complete mitochondrial CR (864 bp) for 133 specimens has been sequenced. 61 variable sites (39 informative for parsimony analysis), 50 transitions, four transversions and nine indels have been detected. C+G composition has been about 36%. 60 haplotypes (Table 1, S1 Table), all of them new (GenBank № OM970166–OM970227), have been revealed. The analysis of 117 concatenated mtDNA *cyt b* and CR sequences (2004 bp), which has included 114 variable sites (71 informative for parsimony analysis), has revealed 68 haplotypes (S1 Table).

Intraspecific genetic structure of the striped field mouse can be studied in the most complete manner based on *cyt b* data (GenBank data provides the most complete coverage of the species range). The analysis of the variable sites’ distribution in *A*. *agrarius cyt b* has revealed that they have been distributed over the entire length of the gene evenly (S1 Fig), and a reduction in the analyzed sequence for every 50 bp has caused a decrease in genetic variation by an average of 4.17% (S3 Table). To avoid the possibility of the results’ distortion due to such decrease, only the complete sequences of *cyt b* have been used in the study.

### Phylogenetic reconstructions

**Fig 2:**
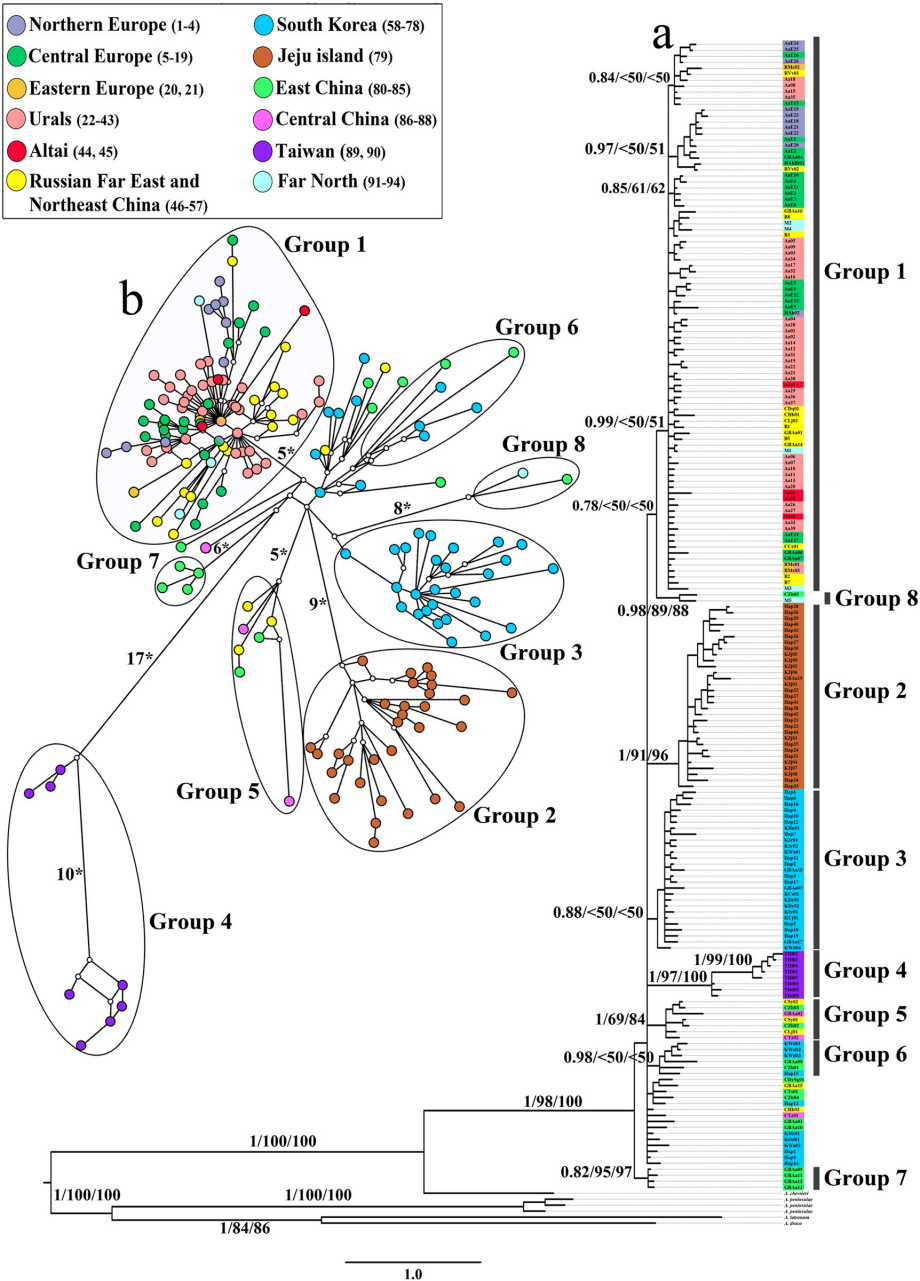
**(A) *A*. *agrarius* phylogenetic tree constructed using the Bayesian analysis on the basis of 192 haplotypes of *cyt b* (1140bp**). Near the branches are the BI>0.70/ML>50/NJ>50 probabilities. **(B) Median-joining network of *cyt b* haplotypes.** Figures near the branches designate the numbers of substitutions. The colors correspond to those given on the Fig 1.

The BI phylogenetic tree of 192 complete *cyt b* haplotypes has demonstrated the presence of eight statistically supported haplogroups (Fig 2A). 15 haplotypes from China and South Korea have not fit into any of the groups. ML and NJ trees have shown the same topology.

The haplotypes from the central part of Northern Eurasia belong to the largest and most widespread Group1, which also combines all haplotypes from the Western part of the range, a number of haplotypes from the Eastern part (Russian Far East and Northeast China) and almost all haplotypes from the isolate in the Far North (with the exception of one M5). Despite the vast distribution area covering regions with different geological history and physiographic conditions, Group1 does not demonstrate clear internal structure that reflects the geographical localization of haplotypes (Fig 2A).

Seven haplogroups (Group 2–8) have been formed from haplotypes from the Eastern part of the species range (Fig 2A). *A*. *agrarius* of islands Jeju and Taiwan (Group 2 and Group 4, respectively) with the latter divided into two subgroups have been the most differentiated. The other four groups have been formed from haplotypes from mainland populations, including shelf islands of the Korean Peninsula: Group 3 is composed of South Korea, Group 5 is composed of Northeast, East and Central China, Group 6 is composed of East China and the shelf islands, and Group 7 is composed of East China (Zhejiang Province). Group 8 is represented by only two haplotypes (Fig 2A), one of which has been found in two individuals in eastern and central China (localities 81, 86 (Fig 1)), and the second is widely distributed in the Far North *A*. *agrarius* isolate (localities 91, 93, 94 (Fig 1)).

The differentiation of the striped field mouse revealed in the phylogenetic reconstructions based on BI has been confirmed by the MJ network of *cyt b* haplotypes (Fig 2B). Eight haplogroups have diverged that correspond to Group 1 –Group 8 of the Bayesian tree (Fig 2A). Within Group 1, the distribution of haplotypes does not shown a clear connection with their geographical localization; haplotypes from the central part of Northern Eurasia (the Urals and Western Siberia) and Eastern Europe occupy positions close to the basal one, and haplotypes from the Far East of Russia and China (Eastern part of the range) are situated at distal positions and are significantly removed from each other (Fig 2B). Among the Eastern haplogroups the most differentiated group (and the most distant from the network center) is Group 4 (Taiwan), consisting of two subgroups. Most of the 15 Chinese and Korean haplotypes, weakly differentiated at the Bayesian tree (Fig 2A), are grouped together at the MJ network (Fig 2B) and are closest to Group 6 (Eastern China and shelf islands of the Korean Peninsula).

The mean genetic distances between haplogroups of *cyt b* have shown that the mainland populations of *A*. *agrarius* have been closer to each other than to populations from Jeju and Taiwan (Table 2). The mean distances between continental haplogroups have been relatively close and have significantly exceeded the distances within haplogroups.

**Table 2:** Mean genetic distances within and between *A*. *agrarius* haplogroups differentiated by *cyt b*.

Group		Mainland groups					Jeju	Taiwan
		1	3	5	6	7	2	4
**Mainland groups**	**1**	5.60/0.79	2.56	2.71	2.70	2.91	3.29	4.20
	**3**	13.0	4.86/0.73	2.79	2.65	2.85	3.30	4.51
	**5**	15.0	13.5	6.36/1.49	2.63	3.03	3.48	4.33
	**6**	14.6	12.8	13.9	8.14/1.64	2.51	3.37	4.32
	**7**	13.8	13.1	13.5	11.4	1.76/0.94	3.61	4.24
**Jeju**	**2**	20.0	18.5	18.9	18.6	18.5	7.34/1.15	4.74
**Taiwan**	**4**	28.9	28.7	27.1	28.3	26.4	31.0	8.53/1.90

under diagonal–mean between group distances (×10^−3^); above diagonal–S.E. (×10^−3^); bias–mean within group distance / S.E (×10^−3^)

Because of low amount of data from the Eastern part of the species range (Table 1), the phylogenetic reconstructions for the CR and concatenated sequences mainly cover the distribution area of Group 1. Since the analysis of concatenated sequences is the most informative, the results of the phylogenetic reconstructions based on CR have not been considered in detail.

**Fig 3:**
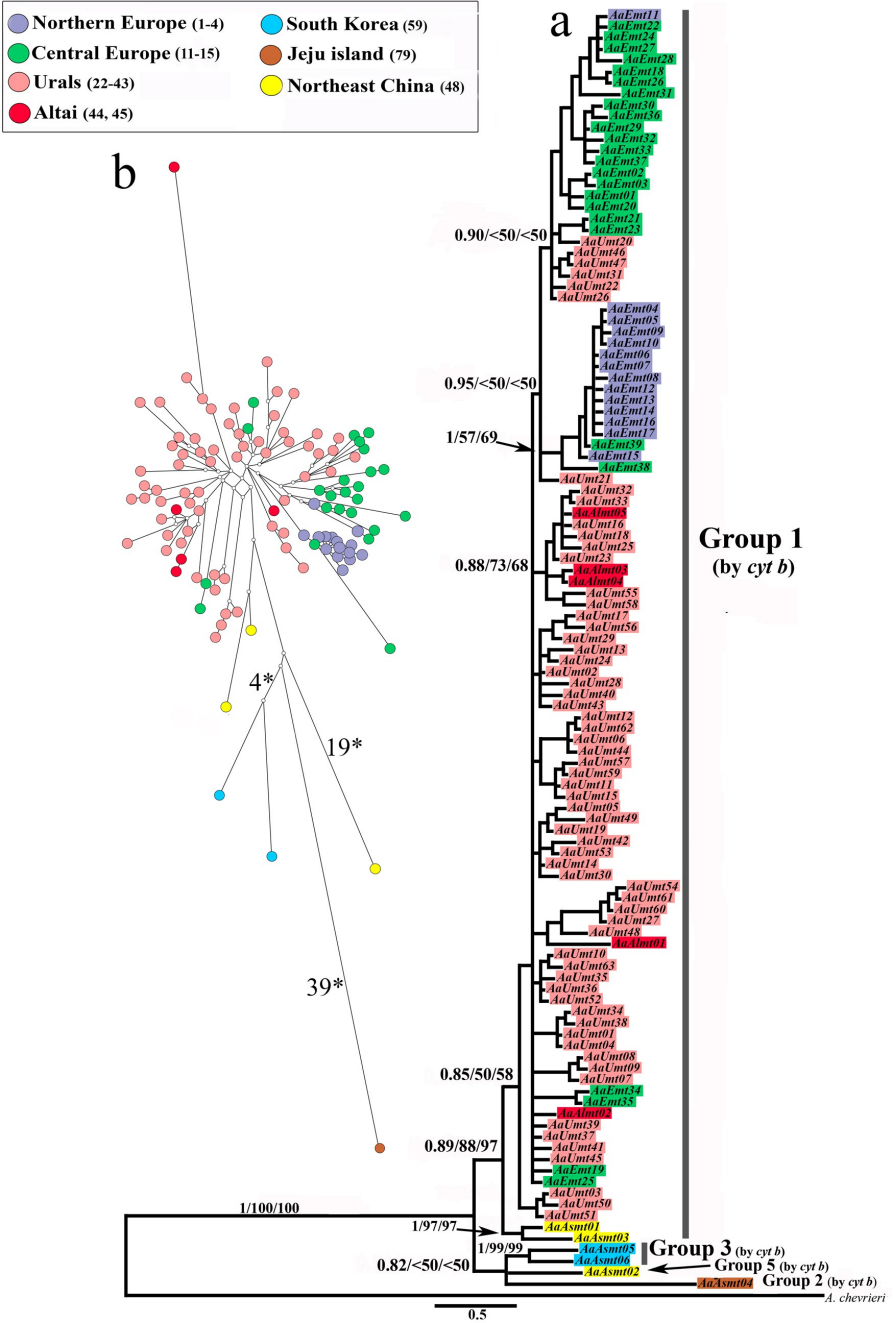
**(A) *A*. *agrarius* phylogenetic tree constructed using the Bayesian analysis on the basis of 113 haplotypes of concatenated sequences (2004 bp).** Near the branches are the BI>0.80/MI>50/NJ>50 probabilities. **(B) Median–joining network of haplotypes.** Figures near branches designate the numbers of substitutions. The colors correspond to those given on the Fig 1.

On the phylogenetic tree of concatenated sequences, the haplotype groups corresponding to Group 1 and Group 3 according to *cyt b* have been distinguished (Fig 3A). Halotypes lying separately belong to Group 2 and Group 5 according to *cyt b*. Therefore, these group names have been used in the further description of the results.

Group 1 contains all haplotypes from the Western part of the range, including those described by us, and two haplotypes of specimens from Northeastern China (Fig 3A), which, according to *cyt b* data, also belong to this group. Despite the presence of statistically supported subgroups, the phylogeographic structure of the Group per se is not clear. The phylogeographic signal has only been observed in the case of the separation of the two haplotypes from China and in the grouping of haplotypes from Germany and Northern Europe (localities 1–4, 8 (Fig 1)), which is confirmed by their locations within Group 1 on the MJ network (Fig 3B). In some cases, the subgroups have included haplotypes from regions geographically distant from each other: for example, the haplotypes from the Urals and Northern and Central Europe, or the haplotypes from the Altai and the Ural regions.

The analysis of within-group genetic distances of the concatenated sequences of Group 1 has not revealed any difference between the Western part of the range and its individual geographic regions (Table 3), which has confirmed the absence of a clear phylogeographic structure.

**Table 3:** Within-group genetic distances of concatenated sequences of *A*. *agrarius*.

Data	N	Mean genetic distance (×10^−3^)	S.E. (×10^−3^)
All data	113	7.49	0.851
Group 1	109	6.64	0.860
Western part of the range	107	6.51	0.842
Europe	39	5.70	1.08
Urals and Western Siberia	63	5.68	0.766
Altai	5	6.77	1.21

#### Genetic diversity and demographic analysis

The indices of haplotype diversity have in general been similar for the Eastern and Western parts of the range. Nucleotide diversity has been higher in the Eastern part of the range (Table 4). The indices of genetic diversity of the haplogroups have not substantially differed except the lower indices’ values for *cyt b* in Group 7 (represented by only one population from Zhejiang Province, China) and Group 8 (Nh = 2).

**Table 4:** The genetic diversity indices and values of selective neutrality test for Eastern and Western parts of the range, and for each haplogroup of *A*. *agrarius*.

Marker	Data	N	*Nh*	*h* ± SD	π ± SD	*k*	*Fs*
***cyt b***	All range	503	192	0.979± 0.003	0.013±0.006	14.78	-23.54*
	Western part	212	73	0.968± 0.008	0.005±0.003	5.92	-24.88*
	Eastern part	291	119	0.954± 0.002	0.015±0.008	17.49	-23.63*
	Group 1	279	92	0.973± 0.004	0.006±0.003	6.34	-24.64*
	Group 2	65	31	0.961±0.010	0.007±0.003	7.55	-9.61*
	Group 3	33	27	0.989±0.010	0.004±0.002	5.10	-21.90*
	Group 4	17	8	0.897±0.042	0.007±0.004	8.12	1.80
	Group 5	8	7	0.964±0.077	0.006±0.004	6.68	-1.26
	Group 6	12	6	0.803±0.096	0.006±0.003	6.52	1.76
	Group 7	9	4	0.694±0.147	0.001±0.001	1.06	-0.93
	Group8	56	2	0.070±0.046	0.0005±0.001	0.56	2.28
***cyt b* + CR**	All range	200	113	0.987±0.003	0.007±0.004	14.46	-23.87*
	Western part	194	107	0.986±0.003	0.007±0.003	13.34	-23.94*
	Eastern part	6	6	1.000±0.096	0.019±0.010	35.60	0.71
	Group 1	196	109	0.986±0.003	0.007±0.003	12.61	-23.93*

N–number of sequences; *Nh*–number of haplotypes; *h*–haplotype diversity; π–nucleotide diversity; *k*–mean number of pairwise differences; SD–standard deviation; *Fs*–values of Fu’s test of selective neutrality

*P≤0.05

For both parts of the *A*. *agrarius* range and for most haplogroups the deviations from neutrality have been significant (taking into account the Fu’s values sign and probability) (Table 4), with the exception of Group 4 (Taiwan), Group 6 (Northeastern China) and Group 8 (the isolate in the Far North) in case of *cyt b*, and of the Eastern part of the range in case of the concatenated sequences (perhaps this is because of the extremely small sample size).

### Demographic history of *A*. *agrarius* in the Western part of the range

**Fig 4:**
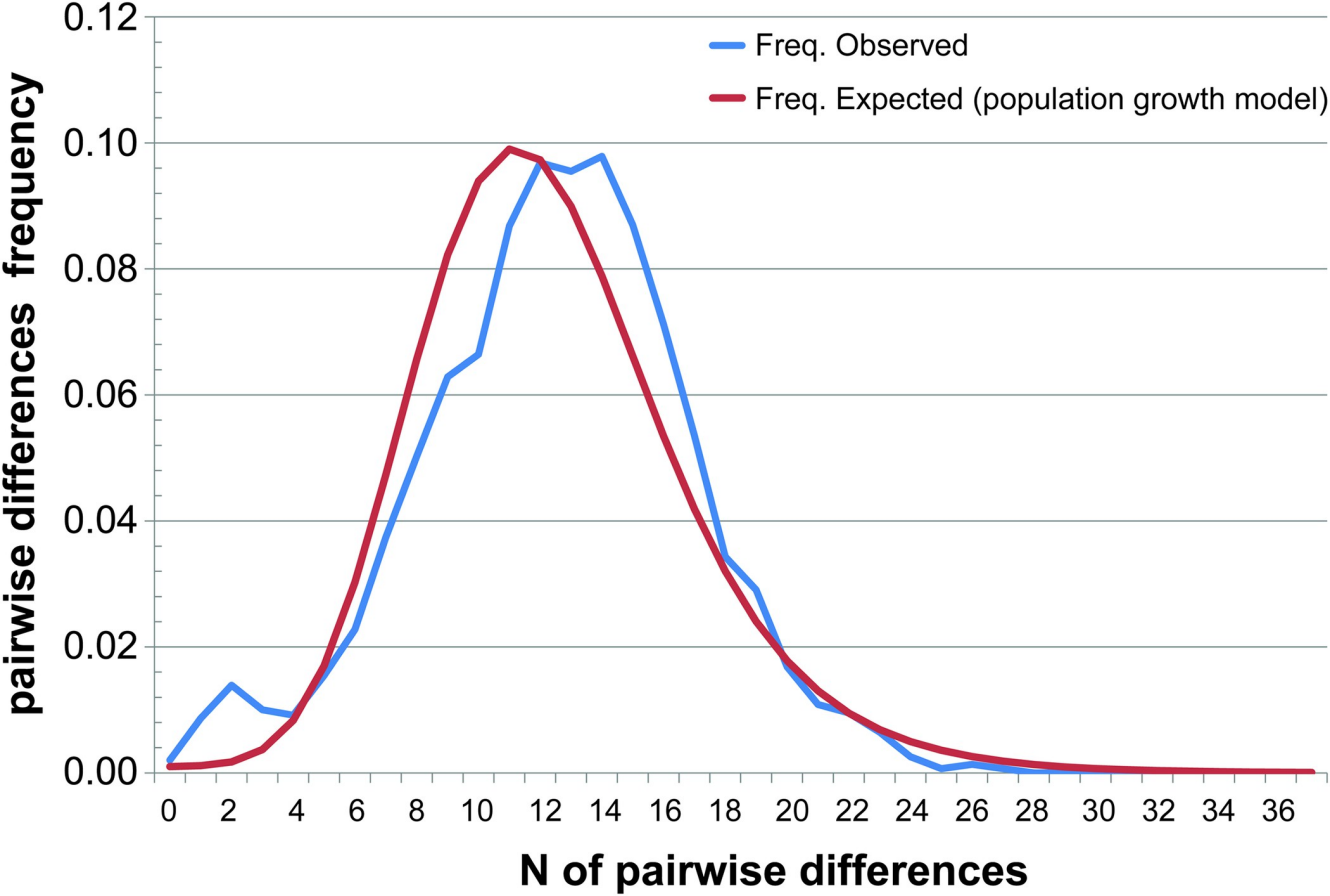
Mismatch distribution analysis for concatenated sequences of *A*. *agrarius* from the Western part of the range.

An analysis of pairwise differences of the concatenated sequences from the Western part of the range (Fig 4) has shown that *A*. *agrarius* has gone through a stage of increasing the effective population size on this territory, which has also been confirmed by the results of the Fu’s neutrality test (Table 4). According to our reconstructions (tMRCA), the lifetime of the common ancestor of concatenated sequences of Group 1 has been about 17.7 [95% HPD 13.2–22.5] kyr, i.e. it has occurred at the final stages of LGM.

## Discussion

Genetic studies of the field mouse using data on the nucleotide sequences of mitochondrial markers have shown that the modern intraspecific genetic structure and, consequently, the evolutionary history of the species are associated with the dynamics of the physiographic conditions of the individual regions during the Quaternary against the background of the global climate change [14, 16–18]. However, the central part of Northern Eurasia has remained practically unexplored, although it has been shown that the Urals, Altai and Western Siberia have played an important role in the modern genetic structure formation for a number of small mammalian species [40–43].

In the study presented, the data on mtDNA variability of *A*. *agrarius* from 23 localities from the central part of Northern Eurasia has been obtained. The phylogenetic reconstructions based on the complete *cyt b* sequences with the inclusion of the new data have shown the division of the species into eight haplogroups (Fig 2). 15 haplotypes from China and South Korea have not been assigned to any group. The two most differentiated groups have been formed by the populations from the Jeju and Taiwan Islands (Group 2 and Group 4), whose evolutionary history has been reviewed in detail previously [9, 17]. The other six haplogroups (Group 1, 3, 5–8) have been represented by mainland populations, indicating a more complex genetic structure *A*. *agrarius* than recent studies have shown [18]. The results of the analysis of the CR and the concatenated sequences of both mitochondrial markers (*cyt b* and CR) have also supported the existence of genetic heterogeneity in the striped field mouse species within the mainland part of the range.

All lineages have been represented in the Eastern part of the range, while in the Western part, only Group 1 has been present. If we take into account the results of the genetic diversity analysis and the demographic history of *A*. *agrarius*, this indicates that the origin of the species and the formation of its genetic diversity have occurred in the Eastern Palearctic. The differentiation of the genetic lines has probably occurred at about the same time, as revealed by the structure of the MJ network of *cyt b* haplotypes (Fig 2B) and similar values of genetic distances between mainland haplogroups (Table 2). According to the results of the genetic variability and demographic analysis (Table 4), the increase in the effective population size (expansion) of *A*. *agrarius* has occurred not only in the Western direction, but also within the Eastern Palearctic.

*A*. *agrarius* has probably colonized the Western Palearctic from the Far East and/or northeastern China [15, 16, 18], which is confirmed by the clustering of haplotypes from these territories in a single group (Group 1 (Figs 2 and 3)). The distribution of eastern haplotypes within Group1 (distant from each other and occupying distal positions) on MJ networks (Figs 2B and 3B) does not allow us to consider them as ancestral haplotypes. Weak differentiation within a vast area of distribution and the results of the analysis of genetic diversity and demographic structure (Table 4, Fig 4) indicate a relatively recent dispersal of the group over the territory occupied today.

According to our reconstructions, the common ancestor lifetime (tMRCA) of all concatenated sequences of Group 1 has been 17.7 [95% HPD 13.2–22.5] kyr, i.e. it can be assumed that the transcontinental expansion has most likely occurred after the LGM with the rising of climatic conditions optimal for the formation of forest vegetation in a considerably large area of Northern Eurasia. This assumption has been confirmed by the earlier hypotheses about the appearance of *A*. *agrarius* in the Western Palearctic at the boundary of the Pleistocene–Holocene or in the Holocene [18, 44, 45], and by the paleontological finds in Europe and the Trans-Urals [46–49]. Fossil finds from the Late Pleistocene deposits of Europe [50, 51] in the context of the Pleistocene–Holocene expansion of the modern *A*. *agrarius* and the Early Pleistocene origin of the species in Southeast Asia (2.68 Mya [23] or 1.7–0.8 Mya [14]) could be evidences of the repeated expansion into the Europe of their ancestral forms. Differentiation of the mainland populations of *A*. *agrarius* in Northeast Asia has presumably occurred 500–175 kyr [14, 16]. It can be assumed that the European expansion of the ancestral form of the Pleistocene mouse, which has not survived the Late Pleistocene—Holocene boundary [50, 51], has also occurred in the same time interval. Perhaps the latest time such a range expansion could have occurred is about 123 kyr in MIS 5e (peak of Eemian interglacial sub-stage, or Ipswichian in Britain), when the climatic conditions for the formation of the forest biome have been even more favorable than in the Middle Holocene or at present [52]. To solve this problem, in addition to well-dated fossils of *Apodemus* from the Pleistocene deposits of Europe, paleoDNA data is needed. This will make it possible to estimate the rate of speciation, both at the genetic and morphological levels.

When reconstructing the history of the *A*. *agrarius*’ range formation in the Western Palearctic, one should account for the similarity of the intragroup genetic distances of Group 1 and of other haplogroups (Table 2) and the lack of differentiation in haplotypes related to their geographic localization, despite the vast territory the Group1 occupies. According to the data from the concatenated sequences, the genetic distances within each of the three regions of the Western part of the species range (Europe, the Urals and Western Siberia, Altai) do not differ from the intragroup distances of the entire Group1 (Table 3) and are close to the genetic distances calculated based on *cyt b* data for other haplogroups (Table 2). The MJ networks (Figs 2B and 3B) have shown that the haplotypes from different regions within Group1 have largely been mixed with each other, and the subgroups of haplotypes we have distinguished most often have no clear ties to single regions.

Given the modern genetic diversity and phylogenetic relationships between the haplotypes from different regions of the Group1 distribution area, the following possible scenarios for its expansion could be suggested:

- the modern genetic diversity of Group1 was formed in the Far East and/or northeastern China, and the fast transcontinental expansion of the genetically diverse group subsequently took place. The disjunction between Eastern and Western parts of the species range followed.

- one lineage of the *A*. *agrarius* from the territory of the Far East or northeastern China entered into the Western Palearctic. Then the formation of a disjunction occurred. Subsequently, the dispersal over the territory occupied by Group1 at present and the formation of modern genetic diversity accompanying by a constant movement of animals within the entire distribution area took place.

Regardless of the scenario, the dispersal of *A*. *agrarius* within the Western part of the modern range could have been facilitated by anthropogenic transformation of landscapes and unintended introduction, leading not only to the occupation of new territories, but also to the movement of animals between already inhabited territories, which has increased genetic drift and prevented isolation and genetic differentiation between populations from geographically distant regions.

## Supporting information

S1 Table List of haplotypes used in phylogeography analysis: Abbreviation, GenBank accession number (access №), location in details, number of the specimens (N), reference. (DOC)

S2 Table List of *cyt b* haplotypes, which together with haplotypes listed in S1 Table, used in variable sites distribution analysis. (DOC)

S3 Table The number (N) and percentage (%) of polymorphic sites of each fragment length 50 bp of *A*. *agrarius cyt b*. Analysis was carried out using 593 complete and partial sequences, including both original and GenBank data (S1 and S2 Tables).
(DOC)

S1 Fig *A*. *agrarius cyt b* polymorphic sites (red color) distribution. (TIF)
