# Supplementary material for: Phylogeography of the striped field mouse (Apodemus agrarius Pallas, 1771) in light of new data from central part of Northern Eurasia
Source: PLoS One. 2022 Oct 20;17(10):e0276466. doi: 10.1371/journal.pone.0276466 (PMC9584417; doi:10.1371/journal.pone.0276466)
Supplement: S1 Table — (DOC) [file pone.0276466.s001.doc]

**S1 Table. List of haplotypes used in phylogeography analysis: abbreviation, GenBank accession number (Access №), location in details, number of the specimens (N), reference**

| **Haplotype**  **(abbrev.)** | **Access №**  **(GenBank)** | **Location** | | | **N** | **Reference** |
| --- | --- | --- | --- | --- | --- | --- |
|  |  | **Localities (map reference)** | **Country** | **Geographical region** |  |  |

| **1** | | **2** | | **3** | | **4** | | **5** | | **6** | | **7** |
| --- | --- | --- | --- | --- | --- | --- | --- | --- | --- | --- | --- | --- |
| *Cyt b* | | | | | | | | | | | | |
| Aa01 | | OM970127 | | Shigaevo village, Sverdlovsk region (36)  Starikova village, Sverdlovsk region (33)  Eastern Urals Reserve, Chelyabinsk region (30) | | Russia | | Urals | | 1  1  1 | | Original data |
| Aa02 | | OM970128 | | Nizhniy Tagil, Sverdlovsk region (40)  Zverinogolovskoye village, Kurgan region (43) | | Russia | | Urals  Western Siberia | | 7  3 | | Original data |
| Aa03 | | OM970129 | | Nizhniy Tagil, Sverdlovsk region (40)  Kinzebulatovo village, Baskortostan (27)  Aytuarskaya Steppe, Orenburg region (25) | | Russia | | Urals | | 2  1  1 | | Original data |
| Aa04 | | OM970130 | | Nizhniy Tagil, Sverdlovsk region (40) | | Russia | | Urals | | 2 | | Original data |
| Aa05 | | OM970131 | | Kharlovskoye village, Sverdlovsk region (37)  Ekaterinburg, Sverdlovsk region (35)  Dvurechensk village, Sverdlovsk region (34)  Starikova village, Sverdlovsk region (33) | | Russia | | Urals | | 1  1  2  3 | | Original data |
| Aa06 | | OM970132 | | Eastern Urals Reserve, Chelyabinsk region (30)  Zverinogolovskoye village, Kurgan region (43) | | Russia | | Urals  Western Siberia | | 2  1 | | Original data |
| Aa07 | | OM970133 | | Eastern Urals Reserve, Chelyabinsk region (30) | | Russia | | Urals | | 3 | | Original data |
| Aa08 | | OM970134 | | Khomutovka village, Sverdlovsk region (38) | | Russia | | Urals | | 5 | | Original data |
| Aa09 | | OM970135 | | Talovskaya Steppe, Orenburg region (24) | | Russia | | Urals | | 2 | | Original data |
| Aa10 | | OM970136 | | Nizhniy Tagil, Sverdlovsk region (40)  Sos'va village, Sverdlovsk region (39) | | Russia | | Urals | | 1  3 | | Original data |
| Aa11 | | OM970137 | | Aytuarskaya Steppe, Orenburg region (25) | | Russia | | Urals | | 4 | | Original data |
| Aa12 | | OM970138 | | Mt. Verbluzhka, Orenburg region (26)  Kinzebulatovo village, Baskortostan (27) | | Russia | | Urals | | 1  1 | | Original data |
| Aa13 | | OM970139 | | Dvurechensk village, Sverdlovsk region (34) | | Russia | | Urals | | 2 | | Original data |
| Aa14 | | OM970140 | | Baturinsky village, Chelyabinsk region (29)  Starikova village, Sverdlovsk region (33)  Dvurechensk village, Sverdlovsk region (34)  Kharlovskoye village, Sverdlovsk region (37)  Sos'va village, Sverdlovsk region (39) | | Russia | | Urals | | 1  1  1  2  4 | | Original data |
| Aa15 | | OM970141 | | Belaya Kholunitsa, Kirov region (22)  Chornaya Kholunitsa village, Kirov region (23)  Kungur, Perm Krai (28) | | Russia | | Urals | | 2  2  1 | | Original data |
| Aa16 | | OM970142 | | Starikova village, Sverdlovsk region (33) | | Russia | | Urals | | 1 | | Original data |
| Aa17 | | OM970143 | | Nizhniy Tagil, Sverdlovsk region (40)  Tomilovo village, Tyumen region (41) | | Russia | | Urals  Western Siberia | | 9  1 | | Original data |
| Aa18 | | OM970144 | | Talovskaya Steppe, Orenburg region (24) | | Russia | | Urals | | 1 | | Original data |
| Aa19 | | OM970145 | | Baturinsky village, Chelyabinsk region (29)  Mashkara village, Tyumen region (42) | | Russia | | Urals  Western Siberia | | 2  1 | | Original data |
| Aa20 | | OM970146 | | Kharlovskoye village, Sverdlovsk region (37)  Mashkara village, Tyumen region (42) | | Russia | | Urals  Western Siberia | | 1  2 | | Original data |
| Aa21 | | OM970147 | | Kharlovskoye village, Sverdlovsk region (37) | | Russia | | Urals | | 2 | | Original data |
| Aa22 | | OM970148 | | Baturinsky village, Chelyabinsk region (29) | | Russia | | Urals | | 2 | | Original data |
| Aa23 | | OM970149 | | Surtayka village, Altai region (45) | | Russia | | Altai | | 4 | | Original data |
| Aa24 | | OM970150 | | Bolshaya Rechka village, Altai region (44)  Surtayka village, Altai region (45) | | Russia | | Altai | | 1  1 | | Original data |
| Aa25 | | OM970151 | | Surtayka village, Altai region (45) | | Russia | | Altai | | 2 | | Original data |
| Aa26 | | OM970152 | | Talovskaya Steppe, Orenburg region (24) | | Russia | | Urals | | 1 | | Original data |
| Aa27 | | OM970153 | | Nizhniy Tagil, Sverdlovsk region (40) | | Russia | | Urals | | 1 | | Original data |
| Aa28 | | OM970154 | | Nizhniy Tagil, Sverdlovsk region (40) | | Russia | | Urals | | 1 | | Original data |
| Aa29 | | OM970155 | | Ilmen Nature Reserve, Chelyabinsk region (31) | | Russia | | Urals | | 1 | | Original data |
| Aa30 | | OM970156 | | Bolshaya Rechka village, Altai region (44) | | Russia | | Altai | | 1 | | Original data |
| Aa31 | | OM970157 | | Ekaterinburg, Sverdlovsk region (35) | | Russia | | Urals | | 2 | | Original data |
| Aa32 | | OM970158 | | Tomilovo village, Tyumen region (41) | | Russia | | Western Siberia | | 1 | | Original data |
| Aa33 | | OM970159 | | Tomilovo village, Tyumen region (41) | | Russia | | Western Siberia | | 1 | | Original data |
| Aa34 | | OM970160 | | Shigaevo village, Sverdlovsk region (36) | | Russia | | Urals | | 2 | | Original data |
| Aa35 | | OM970161 | | Shigaevo village, Sverdlovsk region (36) | | Russia | | Urals | | 1 | | Original data |
| Aa36 | | OM970162 | | Eastern Urals Reserve, Chelyabinsk region (30) | | Russia | | Urals | | 1 | | Original data |
| Aa37 | | OM970163 | | Eastern Urals Reserve, Chelyabinsk region (30) | | Russia | | Urals | | 2 | | Original data |
| Aa38 | | OM970164 | | Eastern Urals Reserve, Chelyabinsk region (30) | | Russia | | Urals | | 4 | | Original data |
| Aa39 | | OM970165 | | Eastern Urals Reserve, Chelyabinsk region (30) | | Russia | | Urals | | 1 | | Original data |
| AaE1 | | KY851959 | | Schleswig-Holstein (8) | | Germany | | Central Europe | | 1 | | [12] |
| AaE2 | | KY851958 | | Schleswig-Holstein (8) | | Germany | | Central Europe | | 1 | | [12] |
| AaE3 | | KY851957 | | Schleswig-Holstein (8) | | Germany | | Central Europe | | 1 | | [12] |
| AaE4 | | KY851956 | | Lower Saxony (7) | | Germany | | Central Europe | | 1 | | [12] |
| AaE5 | | KY851955 | | Lower Saxony (7) | | Germany | | Central Europe | | 1 | | [12] |
| AaE6 | | KY851954 | |  | | Germany | | Central Europe | | 1 | | [12] |
| AaE7 | | KY851953 | | Sachsen-Anhalt (6) | | Germany | | Central Europe | | 1 | | [12] |
| AaE8 | | KY851952 | | Lower Saxony (7) | | Germany | | Central Europe | | 1 | | [12] |
| AaE9 | | KY851951 | | Lower Saxony (7) | | Germany | | Central Europe | | 1 | | [12] |
| AaE10 | | KY851949 | | Lower Saxony (7) | | Germany | | Central Europe | | 1 | | [12] |
| AaE11 | | KY851948 | | Lower Saxony (7) | | Germany | | Central Europe | | 1 | | [12] |
| AaE12 | | KY851947 | | Lower Saxony (7) | | Germany | | Central Europe | | 1 | | [12] |
| AaE13 | | KY851937  KY851945 | | Sacharewo (13)  Bialowieza (15) | | Poland | | Central Europe | | 1  1 | | [12] |
| AaE14 | | KY851944 | | Bialowieza (15) | | Poland | | Central Europe | | 1 | | [12] |
| AaE15 | | KY851940  KY851942 | | Kosy Most (14)  Bialowieza (15) | | Poland | | Central Europe | | 1  2 | | [12] |
| AaE16 | | KY851939 | | Kosy Most (14) | | Poland | | Central Europe | | 1 | | [12] |
| AaE17 | | KY851938 | | Sacharewo (13), Bialowieza (15) | | Poland | | Central Europe | | 2 | | [12] |
| AaE18 | | KY851923, 24, 26-29, 31, 33, 35, 36  KY851897-99, 901  KY851905, 07, 09, 11, 14, 15, 20 | | Lolland Island (2)  Falster Island (3)  Filskov, Central Jutland (4) | | Denmark | | Northern Europe | | 10  4  10 | | [12] |
| AaE19 | | KY851934  KY851895, 896, 900, 903, 904  KY851906, 08, 10, 16, 19, 21 | | Ödeshög (1)  Lolland Island (2)  Falster Island (3)  Filskov, Central Jutland (4) | | Sweden  Denmark | | Northern Europe | | 1  1  9  8 | | [12] |
| AaE20 | | KY851930, 32 | | Lolland Island (2) | | Denmark | | Northern Europe | | 2 | | [12] |
| AaE21 | | KY851925 | | Lolland Island (2) | | Denmark | | Northern Europe | | 3 | | [12] |
| AaE22 | | KY851922 | | Lolland Island (2) | | Denmark | | Northern Europe | | 1 | | [12] |
| AaE23 | | KY851902 | | Falster Island (3) | | Denmark | | Northern Europe | | 1 | | [12] |
| AaE24 | | KY851894 | | Oonurme (11) | | Estonia | | Northern Europe | | 1 | | [12] |
| AaE25 | | KY851893 | | Oonurme (11) | | Estonia | | Northern Europe | | 1 | | [12] |
| AaE26 | | KY851892 | | Leisi (12) | | Estonia | | Northern Europe | | 1 | | [12] |
| B1 | | KU859999 | | Pryluky village, Primorsky Krai (48)  Hasan Reserve, Primorsky Krai (49) | | Russia | | Far East | | 1  3 | | [11] |
| B2 | | KU860001 | | Pryluky village, Primorsky Krai (48)  Krounovka village, Primorsky Krai (50) | | Russia | | Far East | | 3  5 | | [11] |
| B3 | | KU860002 | | Pryluky village, Primorsky Krai (48)  Hasan Reserve, Primorsky Krai (49)  Krounovka village, Primorsky Krai (50)  Kiparisovo village, Primorsky Krai (51) | | Russia | | Far East | | 1  5  3  2 | | [11] |
| B5 | | KU860000 | | Pryluky village, Primorsky Krai (48)  Krounovka village, Primorsky Krai (50) | | Russia | | Far East | | 1  1 | | [11] |
| B7 | | KU860003 | | Pryluky village, Primorsky Krai (48)  Krounovka village, Primorsky Krai (50)  Kiparisovo village, Primorsky Krai (51) | | Russia | | Far East | | 1  4  1 | | [11] |
| B8 | | KU860004 | | Pryluky village, Primorsky Krai 48) | | Russia | | Far East | | 1 | | [11] |
| CCc01 | | KJ082007 | | Changchun (53) | | China | | North-Eastern China | | 1 | | [17] |
| CDq01 | | KJ082011 | | Daqing (54) | | China | | North-Eastern China | | 1 | | [17] |
| CDySg01 | | KJ082019 | | Shouguang, Dongying (80) | | China | | Eastern China | | 7 | | [17] |
| CHb01 | | KJ082012 | | Harbin (55) | | China | | North-Eastern China | | 1 | | [17] |
| CHb02 | | KJ082013 | | Harbin (55) | | China | | North-Eastern China | | 2 | | [17] |
| CLj01 | | KJ082014 | | Longjiang (56) | | China | | North-Eastern China | | 1 | | [17] |
| CLj02 | | KJ082015 | | Longjiang (56) | | China | | North-Eastern China | | 1 | | [17] |
| CSy01 | | KJ082006 | | Shenyang(57) | | China | | North-Eastern China | | 1 | | [17] |
| CSy02 | | KJ082008 | | Shenyang(57) | | China | | North-Eastern China | | 1 | | [17] |
| CTs01 | | KJ082016 | | Taishan (82) | | China | | Eastern China | | 1 | | [17] |
| CTz01 | | KJ082017 | | Tongzi (88) | | China | | Central China | | 1 | | [17] |
| CTz02 | | KJ082018 | | Tongzi (88) | | China | | Central China | | 1 | | [17] |
| CZb01 | | KJ082020 | | Zibo (81) | | China | | Eastern China | | 1 | | [17] |
| CZb02 | | KJ082021 | | Zibo (81) | | China | | Eastern China | | 1 | | [17] |
| CZb03 | | KJ082022 | | Zibo (81) | | China | | Eastern China | | 2 | | [17] |
| CZb04 | | KJ082023 | | Zibo (81) | | China | | Eastern China | | 1 | | [17] |
| CZb05 | | KJ082024, AY389011 | | Zibo (81)  Xian Shaanxi (86) | | China | | Eastern China  Central China | | 1  1 | | [17]  [23] |
| GBAa01 | | AB032851 | | Ussuriysk (46) | | Russia | | Far East | | 1 | | [10] |
| GBAa02 | | AB096809 | | Tongzi (88) | | China | | Central China | | 1 | | [14] |
| GBAa03 | | AB096815 | | Shanghai (84) | | China | | Eastern China | | 1 | | [14] |
| GBAa04 | | AB096817 | | Lubeck (9) | | Germany | | Central Europe | | 1 | | [14] |
| GBAa05 | | AB303225 | | Gansung (68) | | South Korea | | South Korea | | 1 | | [15] |
| GBAa06 | | AB303226 | | Italy (19) | | Italy | | Central Europe | | 1 | | [15] |
| GBAa07 | | AF159390 | | Sremska Mitrovica (18) | | Serbia | | Central Europe | | 1 | | [19] |
| GBAa08 | | AY389012 | | Taishan Shandong (83) | | China | | Eastern China | | 1 | | [23] |
| GBAa09 | | KF294387, 88, 92, 94, 96 | | Longquan, Zhejiang Province (85) | | China | | Eastern China | | 5 | | [26] |
| GBAa10 | | KF294389 | | Longquan, Zhejiang Province (85) | | China | | Eastern China | | 1 | | [26] |
| GBAa11 | | KF294390 | | Longquan, Zhejiang Province (85) | | China | | Eastern China | | 1 | | [26] |
| GBAa12 | | KF294391, 93 | | Longquan, Zhejiang Province (85) | | China | | Eastern China | | 2 | | [26] |
| GBAa13 | | KF294395 | | Longquan, Zhejiang Province (85) | | China | | Eastern China | | 2 | | [26] |
| GBAa14 | | KJ857284 | | Fuyuan, Heilongjiang Province (52) | | China | | North-Eastern China | | 1 | | [25] |
| GBAa15 | | KJ857285 | | Fuyuan, Heilongjiang Province (52) | | China | | North-Eastern China | | 1 | | [25] |
| GBAa16 | | KJ857286 | | Fuyuan, Heilongjiang Province (52) | | China | | North-Eastern China | | 1 | | [25] |
| Hap1 | | HM034868 | | Boreumdo, Incheon (76) | | South Korea | | Korea Islands | | 1 | | [9] |
| Hap2 | | HM034869, 71 | | Cheongdo, Gyeongbuk (69) | | South Korea | | South Korea | | 2 | | [9] |
| Hap3 | | HM034870 | | Cheongdo, Gyeongbuk (69) | | South Korea | | South Korea | | 1 | | [9] |
| Hap4 | | HM034872 | | Cheongdo, Gyeongbuk (69) | | South Korea | | South Korea | | 1 | | [9] |
| Hap5 | | HM034873, 75 | | Yangsan, Gyeongnam (70) | | South Korea | | South Korea | | 2 | | [9] |
| Hap6 | | HM034874 | | Yangsan, Gyeongnam (70) | | South Korea | | South Korea | | 1 | | [9] |
| Hap7 | | HM034876 | | Yangsan, Gyeongnam (70) | | South Korea | | South Korea | | 1 | | [9] |
| Hap8 | | HM034877 | | Yangsan, Gyeongnam (70) | | South Korea | | South Korea | | 1 | | [9] |
| Hap9 | | HM034878 | | Kanghwa, Incheon (77) | | South Korea | | Korea Islands | | 1 | | [9] |
| Hap10 | | HM034879 | | Kanghwa, Incheon (77) | | South Korea | | Korea Islands | | 1 | | [9] |
| Hap11 | | HM034880 | | Samcheok, Kangwon (71) | | South Korea | | South Korea | | 1 | | [9] |
| Hap12 | | HM034881, 82 | | Macheon, Gyeongnam (72) | | South Korea | | South Korea | | 2 | | [9] |
| Hap13 | | HM034883 | | Seokmodo, Incheon (78) | | South Korea | | Korea Islands | | 1 | | [9] |
| Hap14 | | HM034884 | | Seokmodo, Incheon (78) | | South Korea | | Korea Islands | | 1 | | [9] |
| Hap15 | | HM034885-87 | | Seokmodo, Incheon (78) | | South Korea | | Korea Islands | | 3 | | [9] |
| Hap16 | | HM034888 | | Hwacheon, Gangwon (73) | | South Korea | | South Korea | | 1 | | [9] |
| Hap17 | | HM034889 | | Hwacheon, Gangwon (73) | | South Korea | | South Korea | | 1 | | [9] |
| Hap18 | | HM034890 | | Hwacheon, Gangwon (73) | | South Korea | | South Korea | | 1 | | [9] |
| Hap19 | | HM034891 | | Naju, Jeonnam (74) | | South Korea | | South Korea | | 1 | | [9] |
| KJj01 | | HM034892, 924, 36, 40  KJ081981 | | ,Jeju Island (79): Geumak, Hallin, Pyoseon, Seogwipo, Kwaneumsa and Seongpanak | | South Korea | | Jeju Island | | 7 | | [9]  [17] |
| Hap21 | | HM034893 | | Seongsan, Jeju Island (79) | | South Korea | | Jeju Island | | 1 | | [9] |
| KJj09 | | HM034894, 915  KJ081989 | | ,Jeju Island (79): Seongsan, Ara and Gozzawal | | South Korea | | Jeju Island | | 3 | | [9]  [17] |
| Hap23 | | HM034895, 915 | | Seongsan, Jeju Island (79) | | South Korea | | Jeju Island | | 2 | | [9] |
| Hap24 | | HM034896 | | Seongsan, Jeju Island (79) | | South Korea | | Jeju Island | | 1 | | [9] |
| KJj03 | | HM034897, 900  KJ081983 | | Jeju Island (79): Geumak, Ara and Kwaneumsa | | South Korea | | Jeju Island | | 6 | | [9]  [17] |
| Hap26 | | HM034898 | | Jocheon, Jeju Island (79) | | South Korea | | Jeju Island | | 1 | | [9] |
| Hap27 | | HM034899, 909, 10, 27, 30, 33 | | Jeju Island (79): Andeok, Pyoseon and Sanghyo | | South Korea | | Jeju Island | | 6 | | [9] |
| Hap28 | | HM034901, 03, 25 | | Jeju Island (79): Yonggang and Geumak, Hallim | | South Korea | | Jeju Island | | 3 | | [9] |
| Hap29 | | HM034902 | | Yonggang, Jeju Island (79) | | South Korea | | Jeju Island | | 1 | | [9] |
| Hap30 | | HM034904 | | Songdang, Jeju Island (79) | | South Korea | | Jeju Island | | 1 | | [9] |
| Hap31 | | HM034905, 26 | | Sanhyo, Jeju Island (79) | | South Korea | | Jeju Island | | 2 | | [9] |
| Hap32 | | HM034906, 16 | | Jeju Island (79): Sanghyo and Ara | | South Korea | | Jeju Island | | 2 | | [9] |
| Hap33 | | HM034907, 11, 32, 34, 38 | | Pyoseon, Jeju Island (79) | | South Korea | | Jeju Island | | 5 | | [9] |
| Hap34 | | HM034908 | | Pyoseon, Jeju Island (79) | | South Korea | | Jeju Island | | 1 | | [9] |
| Hap35 | | HM034912, 17, 22 | | Ara, Jeju Island (79) | | South Korea | | Jeju Island | | 3 | | [9] |
| Hap36 | | HM034913, 18 | | Ara, Jeju Island (79) | | South Korea | | Jeju Island | | 2 | | [9] |
| Hap37 | | HM034914, 23 | | Ara, Jeju Island (79) | | South Korea | | Jeju Island | | 2 | | [9] |
| Hap38 | | HM034920 | | Yeongpyeong, Jeju Island (79) | | South Korea | | Jeju Island | | 1 | | [9] |
| KJj06 | | HM034921, 29, KJ081986 | | Jeju Island (79): Ara, Seongsan and Gozzawal | | South Korea | | Jeju Island | | 4 | | [9], [17] |
| HAh02 | | KJ082029,  KY851917, 43, 46 | | Abrahamhegy (16)  Kosy Most (14), Bialowieza (15)  Filskov, Central Jutland (4) | | Hungary  Poland  Denmark | | Central Europe  Northern Europe | | 4  3  1 | | [17]  [12] |
| HAhBt01 | | KJ082028 | | Balatonrendes (17) | | Hungary | | Central Europe | | 3 | | [17] |
| HM034866 | | HM034866 | | - | | South Korea | | South Korea | | 1 | | Unpublished |
| GBAa19 | | HM034867 | | Jeju Island (79) | | South Korea | | Jeju Island | | 1 | | Unpublished |
| JN629047 | | JN629047 | | Mt. Jiri (59) | | South Korea | | South Korea | | 1 | | [22] |
| KCa01 | | KJ081996 | | Mt. Chiak (63) | | South Korea | | South Korea | | 1 | | [17] |
| KDy01 | | KJ081994 | | Mt. Deokyu (60) | | South Korea | | South Korea | | 1 | | [17] |
| KDy02 | | KJ081995 | | Mt. Deokyu (60) | | South Korea | | South Korea | | 2 | | [17] |
| KHn01 | | KJ082001 | | Haenam (58) | | South Korea | | South Korea | | 1 | | [17] |
| KJj02 | | KJ081982 | | Jeju Island (79) | | South Korea | | Jeju Island | | 1 | | [17] |
| KJj04 | | KJ081984 | | Jeju Island (79) | | South Korea | | Jeju Island | | 1 | | [17] |
| KJj05 | | KJ081985 | | Jeju Island (79) | | South Korea | | Jeju Island | | 1 | | [17] |
| KJj07 | | KJ081987 | | Jeju Island (79) | | South Korea | | Jeju Island | | 1 | | [17] |
| KJj08 | | KJ081988 | | Jeju Island (79) | | South Korea | | Jeju Island | | 1 | | [17] |
| KJr01 | | KJ081999 | | Mt. Jiri (59) | | South Korea | | South Korea | | 1 | | [17] |
| KMs01 | | KJ081997 | | Munsan (64) | | South Korea | | South Korea | | 1 | | [17] |
| KOd01 | | KJ081998 | | Mt. Odae (65) | | South Korea | | South Korea | | 1 | | [17] |
| KSr01 | | KJ081990 | | Mt. Songri (61) | | South Korea | | South Korea | | 2 | | [17] |
| KSr02 | | KJ081991 | | Mt. Songri (61) | | South Korea | | South Korea | | 1 | | [17] |
| KUj01 | | KJ082000 | | Uljin (66) | | South Korea | | South Korea | | 1 | | [17] |
| KWa01 | | AB303224  KJ081992 | | Yang san (67)  Mt. Weolak (62) | | South Korea | | South Korea | | 1  1 | | [15]  [17] |
| KWa02 | | KJ081993 | | Mt. Weolak (62) | | South Korea | | South Korea | | 3 | | [17] |
| KWd01 | | KJ082002 | | Wan Island (75) | | South Korea | | Korea Islands | | 1 | | [17] |
| KWd02 | | KJ082003 | | Wan Island (75) | | South Korea | | Korea Islands | | 5 | | [17] |
| KWd03 | | KJ082004 | | Wan Island (75) | | South Korea | | Korea Islands | | 1 | | [17] |
| KWd04 | | KJ082005 | | Wan Island (75) | | South Korea | | Korea Islands | | 1 | | [17] |
| M1 | | KR338982 | | Snezhnaya Dolina village, Magadan Region (91) | | Russia | | Far North Isolate | | 1 | | [11] |
| M2 | KR338983 | | Snezhnaya Dolina village, Magadan Region (91) | | Russia | | Far North Isolate | | 1 | | [11] | |
| M3 | KR338984 | | Snezhnaya Dolina village, Magadan Region (91) | | Russia | | Far North Isolate | | 1 | | [11] | |
| M4 | KR338985 | | Talon village, Magadan Region (92) | | Russia | | Far North Isolate | | 23 | | [11] | |
| M5 | KR338986 | | Snezhnaya Dolina village, Magadan Region (91), Snezhny village, Magadan Region (93),  Magadan, Magadan Region (94) | | Russia | | Far North Isolate | | 3  17  34 | | [11] | |
| RMc01 | KJ082026 | | Moscow (20)  Kharlovskoye village, Sverdlovsk region (37)  Khomutovka village, Sverdlovsk region (38)  Eastern Urals Reserve, Chelyabinsk region (30)  Nizhniy Tagil, Sverdlovsk region (40)  Mashkara village, Tyumen region (42) | | Russia | | Eastern Europe  Urals  Western Siberia | | 1  1  1  1  1  1 | | [17]  Original data | |
| RMc02 | KJ082027 | | Moscow (20) | | Russia | | Eastern Europe | | 1 | | [17] | |
| RMc03 | KJ082025 | | Moscow (20)  Talovskaya Steppe, Orenburg region (24)  Ilmen Nature Reserve, Chelyabinsk region (31) | | Russia | | Eastern Europe  Urals | | 1  1  1 | | [17]  Original data | |
| RVv01 | KJ082009 | | Vladivostok, Primorsky Krai (47) | | Russia | | Far East | | 1 | | [17]14 | |
| RVv02 | KJ082010 | | Vladivostok, Primorsky Krai (47) | | Russia | | Far East | | 1 | | [17] | |
| TDd01 | AB096816  KJ082035 | | Dadushan (89) | | Taiwan | | Taiwan | | 3 | | [14]  [17] | |
| TDd02 | KJ082036 | | Dadushan (89) | | Taiwan | | Taiwan | | 1 | | [17] | |
| TDd03 | KJ082037 | | Dadushan (89) | | Taiwan | | Taiwan | | 1 | | [17] | |
| THl01 | KJ082030 | | Hualien (90) | | Taiwan | | Taiwan | | 1 | | [17] | |
| THl02 | KJ082031 | | Hualien (90) | | Taiwan | | Taiwan | | 3 | | [17] | |
| THl03 | KJ082032 | | Hualien (90) | | Taiwan | | Taiwan | | 2 | | [17] | |
| THl04 | KJ082033 | | Hualien (90) | | Taiwan | | Taiwan | | 4 | | [17] | |
| THl05 | KJ082034 | | Hualien (90) | | Taiwan | | Taiwan | | 2 | | [17] | |
| Control region mtDNA | | | | | | | | | | | | |
| CRAa01_U | OM970166 | | Nizhniy Tagil, Sverdlovsk region (40) | | Russia | | Urals | | 5 | | Original data | |
| CRAa02_U | OM970167 | | Nizhniy Tagil, Sverdlovsk region (40)  Talovskaya Steppe, Orenburg region (24) | | Russia | | Urals | | 2  2 | | Original data | |
| CRAa03_U | OM970168 | | Nizhniy Tagil, Sverdlovsk region (40) | | Russia | | Urals | | 2 | | Original data | |
| CRAa04_U | OM970169 | | Belaya Kholunitsa, Kirov region (22)  Starikova village, Sverdlovsk region (33)  Dvurechensk village, Sverdlovsk region (34)  Ekaterinburg, Sverdlovsk region (35)  Kharlovskoye village, Sverdlovsk region (37) | | Russia | | Urals | | 1  3  1  2  1 | | Original data | |
| CRAa05_U | OM970170 | | Eastern Urals Reserve, Chelyabinsk region (30) | | Russia | | Urals | | 3 | | Original data | |
| CRAa06_U | OM970171 | | Eastern Urals Reserve, Chelyabinsk region (30) | | Russia | | Urals | | 3 | | Original data | |
| CRAa07_U | OM970172 | | Ilmen Nature Reserve, Chelyabinsk region (31) | | Russia | | Urals | | 1 | | Original data | |
| CRAa08_U | OM970173 | | Kungur, Perm Krai (28)  Khomutovka village, Sverdlovsk region (38) | | Russia | | Urals | | 1  6 | | Original data | |
| CRAa09_U | OM970174 | | Khomutovka village, Sverdlovsk region (38)  Sos'va village, Sverdlovsk region (39)  Nizhniy Tagil, Sverdlovsk region (40) | | Russia | | Urals | | 1  2  1 | | Original data | |
| CRAa10_U | OM970176 | | Aytuarskaya Steppe, Orenburg region (25) | | Russia | | Urals | | 4 | | Original data | |
| CRAa11_U | OM970177 | | Aytuarskaya Steppe, Orenburg region (25)  Kinzebulatovo village, Baskortostan (27) | | Russia | | Urals | | 1  1 | | Original data | |
| CRAa12_U | OM970178 | | Dvurechensk village, Sverdlovsk region (34) | | Russia | | Urals | | 2 | | Original data | |
| CRAa13_U | OM970179 | | Dvurechensk village, Sverdlovsk region (34) | | Russia | | Urals | | 1 | | Original data | |
| CRAa14_U | OM970180 | | Belaya Kholunitsa, Kirov region (22)  Chornaya Kholunitsa village, Kirov region (23) | | Russia | | Urals | | 1  1 | | Original data | |
| CRAa15_U | OM970181 | | Chornaya Kholunitsa village, Kirov region (23) | | Russia | | Urals | | 1 | | Original data | |
| CRAa16_U | OM970182 | | Starikova village, Sverdlovsk region (33) | | Russia | | Urals | | 1 | | Original data | |
| CRAa17_U | OM970183 | | Mashkara village, Tyumen region (42) | | Russia | | Western Siberia | | 1 | | Original data | |
| CRAa18_U | OM970184 | | Baturinsky village, Chelyabinsk region (29)  Mashkara village, Tyumen region (42) | | Russia | | Urals  Western Siberia | | 2  1 | | Original data | |
| CRAa19_U | OM970185 | | Mashkara village, Tyumen region (42) | | Russia | | Western Siberia | | 1 | | Original data | |
| CRAa20_U | OM970187 | | Mashkara village, Tyumen region (42) | | Russia | | Western Siberia | | 1 | | Original data | |
| CRAa21_U | OM970188 | | Nizhniy Tagil, Sverdlovsk region (40)  Tomilovo village, Tyumen region (41) | | Russia | | Urals  Western Siberia | | 3  1 | | Original data | |
| CRAa22_U | OM970189 | | Sos'va village, Sverdlovsk region (39) | | Russia | | Urals | | 1 | | Original data | |
| CRAa23_U | OM970190 | | Baturinsky village, Chelyabinsk region (29)  Starikova village, Sverdlovsk region (33)  Kharlovskoye village, Sverdlovsk region (37) | | Russia | | Urals | | 1  1  2 | | Original data | |
| CRAa24_U | OM970191 | | Eastern Urals Reserve, Chelyabinsk region (30)  Starikova village, Sverdlovsk region (33)  Shigaevo village, Sverdlovsk region (36) | | Russia | | Urals | | 1  1  2 | | Original data | |
| CRAa25_U | OM970192 | | Kharlovskoye village, Sverdlovsk region (37) | | Russia | | Urals | | 1 | | Original data | |
| CRAa26_U | OM970193 | | Kharlovskoye village, Sverdlovsk region (37) | | Russia | | Urals | | 1 | | Original data | |
| CRAa27_U | OM970194 | | Kharlovskoye village, Sverdlovsk region (37) | | Russia | | Urals | | 1 | | Original data | |
| CRAa28_U | OM970195 | | Kharlovskoye village, Sverdlovsk region (37) | | Russia | | Urals | | 1 | | Original data | |
| CRAa29_U | OM970196 | | Baturinsky village, Chelyabinsk region (29) | | Russia | | Urals | | 1 | | Original data | |
| CRAa30_U | OM970198 | | Sos'va village, Sverdlovsk region (39) | | Russia | | Urals | | 3 | | Original data | |
| CRAa31_U | OM970199 | | Baturinsky village, Chelyabinsk region (29) | | Russia | | Urals | | 1 | | Original data | |
| CRAa32_U | OM970200 | | Mt. Verbluzhka, Orenburg region (26) | | Russia | | Urals | | 1 | | Original data | |
| CRAa33_U | OM970201 | | Zverinogolovskoye village, Kurgan region (43) | | Russia | | Western Siberia | | 3 | | Original data | |
| CRAa34_U | OM970202 | | Talovskaya Steppe, Orenburg region (24) | | Russia | | Urals | | 2 | | Original data | |
| CRAa35_U | OM970203 | | Nizhniy Tagil, Sverdlovsk region (40) | | Russia | | Urals | | 1 | | Original data | |
| CRAa36_U | OM970204 | | Nizhniy Tagil, Sverdlovsk region (40) | | Russia | | Urals | | 3 | | Original data | |
| CRAa37_U | OM970205 | | Nizhniy Tagil, Sverdlovsk region (40) | | Russia | | Urals | | 1 | | Original data | |
| CRAa38_U | OM970206 | | Nizhniy Tagil, Sverdlovsk region (40) | | Russia | | Urals | | 1 | | Original data | |
| CRAa39_U | OM970207 | | Nizhniy Tagil, Sverdlovsk region (40) | | Russia | | Urals | | 1 | | Original data | |
| CRAa40_U | OM970209 | | Ilmen Nature Reserve, Chelyabinsk region (31) | | Russia | | Urals | | 1 | | Original data | |
| CRAa41_U | OM970210 | | Nizhniy Tagil, Sverdlovsk region (40) | | Russia | | Urals | | 1 | | Original data | |
| CRAa42_U | OM970211 | | Nizhniy Tagil, Sverdlovsk region (40) | | Russia | | Urals | | 1 | | Original data | |
| CRAa43_U | OM970212 | | Nizhniy Tagil, Sverdlovsk region (40) | | Russia | | Urals | | 2 | | Original data | |
| CRAa44_U | OM970213 | | Sos'va village, Sverdlovsk region (39) | | Russia | | Urals | | 3 | | Original data | |
| CRAa45_U | OM970214 | | Zverinogolovskoye village, Kurgan region (43) | | Russia | | Western Siberia | | 3 | | Original data | |
| CRAa46_U | OM970215 | | Kungur, Perm Krai (28)  Khomutovka village, Sverdlovsk region (38) | | Russia | | Urals | | 1  1 | | Original data | |
| CRAa47_U | OM970216 | | Eastern Urals Reserve, Chelyabinsk reg. (30, 32)  Ekaterinburg, Sverdlovsk region (35) | | Russia | | Urals | | 2  3 | | Original data | |
| CRAa48_U | OM970217 | | Shigaevo village, Sverdlovsk region (36) | | Russia | | Urals | | 1 | | Original data | |
| CRAa49_U | OM970218 | | Tomilovo village, Tyumen region (41) | | Russia | | Western Siberia | | 1 | | Original data | |
| CRAa50_U | OM970220 | | Shigaevo village, Sverdlovsk region (36) | | Russia | | Urals | | 2 | | Original data | |
| CRAa51_U | OM970221 | | Shigaevo village, Sverdlovsk region (36) | | Russia | | Urals | | 1 | | Original data | |
| CRAa52_U | OM970222 | | Eastern Urals Reserve, Chelyabinsk region (30) | | Russia | | Urals | | 2 | | Original data | |
| CRAa53_U | OM970223 | | Eastern Urals Reserve, Chelyabinsk region (30) | | Russia | | Urals | | 1 | | Original data | |
| CRAa54_U | OM970224 | | Eastern Urals Reserve, Chelyabinsk region (30) | | Russia | | Urals | | 1 | | Original data | |
| CRAa55_U | OM970225 | | Eastern Urals Reserve, Chelyabinsk reg. (30, 32) | | Russia | | Urals | | 1, 2 | | Original data | |
| CRAa56_U | OM970226 | | Eastern Urals Reserve, Chelyabinsk region (30) | | Russia | | Urals | | 1 | | Original data | |
| CRAa57_U | OM970227 | | Eastern Urals Reserve, Chelyabinsk region (30) | | Russia | | Urals | | 1 | | Original data | |
| CRAa1_Alt | OM970175 | | Surtayka village, Altai region (45) | | Russia | | Altai | | 4 | | Original data | |
| CRAa2_Alt | OM970186 | | Surtayka village, Altai region (45) | | Russia | | Altai | | 1 | | Original data | |
| CRAa3_Alt | OM970197 | | Surtayka village, Altai region (45)  Talovskaya Steppe, Orenburg region (24)  Eastern Urals Reserve, Chelyabinsk region (30) | | Russia | | Altai  Urals | | 2  1  4 | | Original data | |
| CRAa4_Alt | OM970208 | | Bolshaya Rechka village, Altai region (44) | | Russia | | Altai | | 1 | | Original data | |
| CRAa5_Alt | OM970219 | | Bolshaya Rechka village, Altai region (44) | | Russia | | Altai | | 1 | | Original data | |
| CRAAs01 | KJ857302 | | Fuyuan, Heilongjiang Province (52) | | China | | North-Eastern China | | 1 | | [25] | |
| CRAAs02 | KJ857301 | | Fuyuan, Heilongjiang Province (52) | | China | | North-Eastern China | | 1 | | [25] | |
| CRAAs03 | KJ857300 | | Fuyuan, Heilongjiang Province (52) | | China | | North-Eastern China | | 1 | | [25] | |
| CRAAs04 | HQ655925 | | Hubei (87) | | China | | Central China | | 1 | | [24] | |
| CRAAs05 | HQ655924 | | Hubei (87) | | China | | Central China | | 1 | | [24] | |
| CRAAs06 | HQ655923 | | Hubei (87) | | China | | Central China | | 1 | | [24] | |
| CRAAs07 | HQ655922 | | Hubei (87) | | China | | Central China | | 1 | | [24] | |
| CRAAs08 | HQ655921 | | Hubei (87) | | China | | Central China | | 1 | | [24] | |
| CRAAs09 | HQ655920 | | Hubei (87) | | China | | Central China | | 1 | | [24] | |
| CRAAs10 | HQ655919 | | Hubei (87) | | China | | Central China | | 1 | | [24] | |
| CRAAs11 | HQ655918 | | Hubei (87) | | China | | Central China | | 1 | | [24] | |
| CRAAs12 | HQ655917 | | Hubei (87) | | China | | Central China | | 1 | | [24] | |
| CRAAs13 | HQ655916 | | Hubei (87) | | China | | Central China | | 1 | | [24] | |
| CRAEu01 | KY851955, 61 | | Lower Saxony (7) | | Germany | | Central Europe | | 2 | | [12] | |
| CRAEu02 | KY851947, 54, 60 | | Lower Saxony (7) | | Germany | | Central Europe | | 4 | | [12] | |
| CRAEu03 | KY851896, 98, 99, 900, 02, 04 ,05, 09-16, 19, 21, 22, 25, 27, 30, 31-34, 59 | | Ödeshög (1)  Falster Island (3)  Filskov, Central Jutland (4)  Lolland Island (2)  Schleswig-Holstein (8) | | Sweden  Denmark  Germany | | Northern Europe  Central Europe | | 1  14  1  1 | | [12] | |
| CRAEu04 | KY851958 | | Schleswig-Holstein (8) | | Germany | | Central Europe | | 1 | | [12] | |
| CRAEu05 | KY851948, 50, 57, | | Lower Saxony (7)  Schleswig-Holstein (8) | | Germany | | Central Europe | | 3  1 | | [12] | |
| CRAEu06 | KY851956 | | Lower Saxony (7) | | Germany | | Central Europe | | 1 | | [12] | |
| CRAEu07 | KY851953 | | Sachsen-Anhalt (6) | | Germany | | Central Europe | | 1 | | [12] | |
| CRAEu08 | KY851952 | | Lower Saxony (7) | | Germany | | Central Europe | | 1 | | [12] | |
| CRAEu09 | KY851951 | | Lower Saxony (7) | | Germany | | Central Europe | | 1 | | [12] | |
| CRAEu10 | KY851949 | | Lower Saxony (7) | | Germany | | Central Europe | | 1 | | [12] | |
| CRAEu11 | KY851946 | | Bialowieza (15) | | Poland | | Central Europe | | 1 | | [12] | |
| CRAEu12 | KY851945 | | Bialowieza (15) | | Poland | | Central Europe | | 1 | | [12] | |
| CRAEu13 | KY851944 | | Bialowieza (15) | | Poland | | Central Europe | | 1 | | [12] | |
| CRAEu14 | KY851943 | | Bialowieza (15) | | Poland | | Central Europe | | 1 | | [12] | |
| CRAEu15 | KY851942 | | Bialowieza (15) | | Poland | | Central Europe | | 2 | | [12] | |
| CRAEu16 | KY851941 | | Kosy Most (14) | | Poland | | Central Europe | | 1 | | [12] | |
| CRAEu17 | KY851940 | | Kosy Most (14) | | Poland | | Central Europe | | 1 | | [12] | |
| CRAEu18 | KY851939 | | Kosy Most (14) | | Poland | | Central Europe | | 1 | | [12] | |
| CRAEu19 | KY851938 | | Sacharewo (13), Bialowieza (15) | | Poland | | Central Europe | | 2 | | [12] | |
| CRAEu20 | KY851937 | | Sacharewo (13) | | Poland | | Central Europe | | 1 | | [12] | |
| CRAEu21 | KY851936 | | Lolland Island (2) | | Denmark | | Northern Europe | | 1 | | [12] | |
| CRAEu22 | KY851935 | | Lolland Island (2) | | Denmark | | Northern Europe | | 1 | | [12] | |
| CRAEu23 | KY851895, 97, 903, 07, 08, 20, 26, 28 ,29 | | Falster Island (3)  Filskov, Central Jutland (4)  Lolland Island (2) | | Denmark | | Northern Europe | | 3  4  3 | | [12] | |
| CRAEu24 | KY851923, 24 | | Lolland Island (2) | | Denmark | | Northern Europe | | 2 | | [12] | |
| CRAEu25 | KY851918 | | Filskov, Central Jutland (4) | | Denmark | | Northern Europe | | 1 | | [12] | |
| CRAEu26 | KY851917 | | Filskov, Central Jutland (4) | | Denmark | | Northern Europe | | 1 | | [12] | |
| CRAEu27 | KY851906 | | Filskov, Central Jutland (4) | | Denmark | | Northern Europe | | 2 | | [12] | |
| CRAEu28 | KY851901 | | Falster Island (3) | | Denmark | | Northern Europe | | 1 | | [12] | |
| CRAEu29 | KY851893, 94 | | Oonurme (11) | | Estonia | | Northern Europe | | 2 | | [12] | |
| CRAEu30 | KY851892 | | Leisi (12) | | Estonia | | Northern Europe | | 1 | | [12] | |
| CRAEu32 | AY588250 | | Krasna Lipa (10) | | Czech Republic | | Central Europe | | 1 | | [20] | |
| CRAEu32 | EU188456 | | Lipetsk (21) | | Russia | | Eastern Europe | | 1 | | [21] | |
| CRAAs14 | HM034866 | | - | | South Korea | | South Korea | | 1 | | Unpublished | |
| CRAAs15 | HM034867 | | Jeju Island (79) | | South Korea | | Jeju Island | | 1 | | Unpublished | |
| CRAAs16 | JN629047 | | Mt. Jiri (55) | | South Korea | | South Korea | | 1 | | [22] | |
| Concatenated sequences (*cyt b* + CR) | | | | | | | | | | | | |
| AaAlmt01 | OM970149 + OM970175 | | Surtayka village, Altai region (45) | | Russia | | Altai | | 4 | | Original data | |
| AaAlmt02 | OM970156 + OM970208 | | Bolshaya Rechka village, Altai region (44) | | Russia | | Altai | | 1 | | Original data | |
| AaAlmt03 | OM970150 + OM970219 | | Bolshaya Rechka village, Altai region (44) | | Russia | | Altai | | 1 | | Original data | |
| AaAlmt04 | OM970150 + OM970186 | | Surtayka village, Altai region (45) | | Russia | | Altai | | 1 | | Original data | |
| AaAlmt05 | OM970151 + OM970197 | | Surtayka village, Altai region (45) | | Russia | | Altai | | 2 | | Original data | |
| AaEmt01 | KY851892 | | Leisi (12) | | Estonia | | Northern Europe | | 1 | | [12] | |
| AaEmt02 | KY851893KY851892 | | Oonurme (11) | | Estonia | | Northern Europe | | 1 | | [12] | |
| AaEmt03 | KY851894 | | Oonurme (11) | | Estonia | | Northern Europe | | 1 | | [12] | |
| AaEmt04 | KY851895, 903, 908 | | Falster Island (3) | | Denmark | | Northern Europe | | 3 | | [12] | |
| AaEmt05 | KY851896  KY851900, 904  KY851910, 916, 919, 921  KY851924 | | Ödeshög (1)  Falster Island (3)  Filskov, Central Jutland (4)  Lolland Island (2) | | Sweden  Denmark | | Northern Europe | | 1  7  5  1 | | [12] | |
| AaEmt06 | KY851897  KY851907, 920  KY851926, 928, 929 | | Falster Island (3)  Filskov, Central Jutland (4)  Lolland Island (2) | | Denmark | | Northern Europe | | 1  3  3 | | [12] | |
| AaEmt07 | KY851898, 899  KY851905, 909, 911, 914, 915  KY851927, 931, 933 | | Falster Island (3)  Filskov, Central Jutland (4)  Lolland Island (2) | | Denmark | | Northern Europe | | 2  7  3 | | [12] | |
| AaEmt08 | KY851901 | | Falster Island (3) | | Denmark | | Northern Europe | | 1 | | [12] | |
| AaEmt09 | KY851902 | | Falster Island (3) | | Denmark | | Northern Europe | | 1 | | [12] | |
| AaEmt10 | KY851906 | | Filskov, Central Jutland (4) | | Denmark | | Northern Europe | | 2 | | [12] | |
| AaEmt11 | KY851917 | | Filskov, Central Jutland (4) | | Denmark | | Northern Europe | | 1 | | [12] | |
| AaEmt12 | KY851922 | | Lolland Island (2) | | Denmark | | Northern Europe | | 1 | | [12] | |
| AaEmt13 | KY851923, 924 | | Lolland Island (2) | | Denmark | | Northern Europe | | 2 | | [12] | |
| AaEmt14 | KY851925 | | Lolland Island (2) | | Denmark | | Northern Europe | | 1 | | [12] | |
| AaEmt15 | KY851930, 932 | | Lolland Island (2) | | Denmark | | Northern Europe | | 2 | | [12] | |
| AaEmt16 | KY851935 | | Lolland Island (2) | | Denmark | | Northern Europe | | 1 | | [12] | |
| AaEmt17 | KY851936 | | Lolland Island (2) | | Denmark | | Northern Europe | | 1 | | [12] | |
| AaEmt18 | KY851937 | | Sacharewo (13) | | Poland | | Central Europe | | 1 | | [12] | |
| AaEmt19 | KY851938 | | Sacharewo (13), Bialowieza (15) | | Poland | | Central Europe | | 2 | | [12] | |
| AaEmt20 | KY851939 | | Kosy Most (14) | | Poland | | Central Europe | | 1 | | [12] | |
| AaEmt21 | KY851940 | | Kosy Most (14) | | Poland | | Central Europe | | 1 | | [12] | |
| AaEmt22 | KY851941 | | Kosy Most (14) | | Poland | | Central Europe | | 1 | | [12] | |
| AaEmt23 | KY851942 | | Bialowieza (15) | | Poland | | Central Europe | | 2 | | [12] | |
| AaEmt24 | KY851943 | | Bialowieza (15) | | Poland | | Central Europe | | 1 | | [12] | |
| AaEmt25 | KY851944 | | Bialowieza (15) | | Poland | | Central Europe | | 1 | | [12] | |
| AaEmt26 | KY851945 | | Bialowieza (15) | | Poland | | Central Europe | | 1 | | [12] | |
| AaEmt27 | KY851946 | | Bialowieza (15) | | Poland | | Central Europe | | 1 | | [12] | |
| AaEmt28 | KY851947 | | Lower Saxony (7) | | Germany | | Central Europe | | 1 | | [12] | |
| AaEmt29 | KY851948 | | Lower Saxony (7) | | Germany | | Central Europe | | 1 | | [12] | |
| AaEmt30 | KY851949 | | Lower Saxony (7) | | Germany | | Central Europe | | 1 | | [12] | |
| AaEmt31 | KY851951 | | Lower Saxony (7) | | Germany | | Central Europe | | 1 | | [12] | |
| AaEmt32 | KY851952 | | Lower Saxony (7) | | Germany | | Central Europe | | 1 | | [12] | |
| AaEmt33 | KY851953 | | Sachsen-Anhalt (6) | | Germany | | Central Europe | | 1 | | [12] | |
| AaEmt34 | KY851954 | | - | | Germany | | Central Europe | | 1 | | [12] | |
| AaEmt35 | KY851955 | | Lower Saxony (7) | | Germany | | Central Europe | | 1 | | [12] | |
| AaEmt36 | KY851956 | | Lower Saxony (7) | | Germany | | Central Europe | | 1 | | [12] | |
| AaEmt37 | KY851957 | | Schleswig-Holstein (8) | | Germany | | Central Europe | | 1 | | [12] | |
| AaEmt38 | KY851958 | | Schleswig-Holstein (8) | | Germany | | Central Europe | | 1 | | [12] | |
| AaEmt39 | KY851959 | | Schleswig-Holstein (8) | | Germany | | Central Europe | | 1 | | [12] | |
| AaAsmt01 | KJ857284 + KJ857300 | | Fuyuan, Heilongjiang Province (48) | | China | | North-Eastern China | | 1 | | [25] | |
| AaAsmt02 | KJ857285 + KJ857301 | | Fuyuan, Heilongjiang Province (48) | | China | | North-Eastern China | | 1 | | [25] | |
| AaAsmt03 | KJ857286 + KJ857302 | | Fuyuan, Heilongjiang Province (48) | | China | | North-Eastern China | | 1 | | [25] | |
| AaUmt01 | OM970147 + OM970192 | | Kharlovskoye village, Sverdlovsk region (37) | | Russia | | Urals | | 1 | | Original data | |
| AaUmt02 | KJ082026 + OM970193 | | Kharlovskoye village, Sverdlovsk region (37) | | Russia | | Urals | | 1 | | Original data | |
| AaUmt03 | OM970146 + OM970194 | | Kharlovskoye village, Sverdlovsk region (37) | | Russia | | Urals | | 1 | | Original data | |
| AaUmt04 | OM970147 + OM970195 | | Kharlovskoye village, Sverdlovsk region (37) | | Russia | | Urals | | 1 | | Original data | |
| AaUmt05 | OM970131 + OM970169 | | Starikova village, Sverdlovsk region (33)  Dvurechensk village, Sverdlovsk region (34)  Kharlovskoye village, Sverdlovsk region (37) | | Russia | | Urals | | 2  3  1 | | Original data | |
| AaUmt06 | OM970140 + OM970190 | | Baturinsky village, Chelyabinsk region (29)  Starikova village, Sverdlovsk region (33)  Kharlovskoye village, Sverdlovsk region (37) | | Russia | | Urals | | 1  1  2 | | Original data | |
| AaUmt07 | OM970145 + OM970184 | | Baturinsky village, Chelyabinsk region (29)  Mashkara village, Tyumen region (42) | | Russia | | Urals  Western Siberia | | 2  1 | | Original data | |
| AaUmt08 | OM970148 + OM970196 | | Baturinsky village, Chelyabinsk region (29) | | Russia | | Urals | | 1 | | Original data | |
| AaUmt09 | OM970148 + OM970199 | | Baturinsky village, Chelyabinsk region (29) | | Russia | | Urals | | 1 | | Original data | |
| AaUmt10 | OM970136 + OM970174 | | Sos'va village, Sverdlovsk region (39)  Nizhniy Tagil, Sverdlovsk region (40) | | Russia | | Urals | | 2  1 | | Original data | |
| AaUmt11 | OM970128 + OM970166 | | Nizhniy Tagil, Sverdlovsk region (40) | | Russia | | Urals | | 5 | | Original data | |
| AaUmt12 | OM970140 + OM970198 | | Sos'va village, Sverdlovsk region (39) | | Russia | | Urals | | 3 | | Original data | |
| AaUmt13 | OM970138 + OM970200 | | Mt. Verbluzhka, Orenburg region (26) | | Russia | | Urals | | 1 | | Original data | |
| AaUmt14 | OM970129 + OM970167 | | Nizhniy Tagil, Sverdlovsk region (40) | | Russia | | Urals | | 2 | | Original data | |
| AaUmt15 | OM970128 + OM970201 | | Zverinogolovskoye village, Kurgan region (43) | | Russia | | Western Siberia | | 3 | | Original data | |
| AaUmt16 | OM970132 + OM970214 | | Zverinogolovskoye village, Kurgan region (43) | | Russia | | Western Siberia | | 1 | | Original data | |
| AaUmt17 | OM970130 + OM970168 | | Nizhniy Tagil, Sverdlovsk region (40) | | Russia | | Urals | | 2 | | Original data | |
| AaUmt18 | OM970152 + OM970168 | | Talovskaya Steppe, Orenburg region (24) | | Russia | | Urals | | 1 | | Original data | |
| AaUmt19 | OM970135 + OM970167 | | Talovskaya Steppe, Orenburg region (24) | | Russia | | Urals | | 2 | | Original data | |
| AaUmt20 | OM970144 + OM970202 | | Talovskaya Steppe, Orenburg region (24) | | Russia | | Urals | | 1 | | Original data | |
| AaUmt21 | KJ082025 + OM970202 | | Talovskaya Steppe, Orenburg region (24) | | Russia | | Urals | | 1 | | Original data | |
| AaUmt22 | OM970134 + OM970173 | | Khomutovka village, Sverdlovsk region (38) | | Russia | | Urals | | 5 | | Original data | |
| AaUmt23 | OM970132 + OM970170 | | Eastern Urals Reserve, Chelyabinsk region (30) | | Russia | | Urals | | 2 | | Original data | |
| AaUmt24 | OM970157 + OM970216 | | Ekaterinburg, Sverdlovsk region (35) | | Russia | | Urals | | 1 | | Original data | |
| AaUmt25 | OM970133 + OM970171 | | Eastern Urals Reserve, Chelyabinsk region (30) | | Russia | | Urals | | 3 | | Original data | |
| AaUmt26 | OM970141 + OM970173 | | Kungur, Perm Krai (28) | | Russia | | Urals | | 1 | | Original data | |
| AaUmt27 | OM970143 + OM970188 | | Nizhniy Tagil, Sverdlovsk region (40)  Tomilovo village, Tyumen region (41) | | Russia | | Urals  Western Siberia | | 3  1 | | Original data | |
| AaUmt28 | OM970159 + OM970218 | | Tomilovo village, Tyumen region (41) | | Russia | | Western Siberia | | 1 | | Original data | |
| AaUmt29 | OM970127 + OM970191 | | Starikova village, Sverdlovsk region (33)  Shigaevo village, Sverdlovsk region (36) | | Russia | | Urals | | 1  1 | | Original data | |
| AaUmt30 | OM970160 + OM970220 | | Shigaevo village, Sverdlovsk region (36) | | Russia | | Urals | | 2 | | Original data | |
| AaUmt31 | OM970161 + OM970221 | | Shigaevo village, Sverdlovsk region (36) | | Russia | | Urals | | 1 | | Original data | |
| AaUmt32 | OM970162 + OM970197 | | Eastern Urals Reserve, Chelyabinsk region (30) | | Russia | | Urals | | 1 | | Original data | |
| AaUmt33 | OM970163 + OM970168 | | Eastern Urals Reserve, Chelyabinsk region (30) | | Russia | | Urals | | 2 | | Original data | |
| AaUmt34 | OM970164 + OM970222 | | Eastern Urals Reserve, Chelyabinsk region (30) | | Russia | | Urals | | 2 | | Original data | |
| AaUmt35 | KJ082025 + OM970172 | | Ilmen Nature Reserve, Chelyabinsk region (31) | | Russia | | Urals | | 1 | | Original data | |
| AaUmt36 | KJ082026 + OM970174 | | Khomutovka village, Sverdlovsk region (38) | | Russia | | Urals | | 1 | | Original data | |
| AaUmt37 | KJ082026 + OM970203 | | Nizhniy Tagil, Sverdlovsk region (40) | | Russia | | Urals | | 1 | | Original data | |
| AaUmt38 | OM970164 + OM970224 | | Eastern Urals Reserve, Chelyabinsk region (30) | | Russia | | Urals | | 1 | | Original data | |
| AaUmt39 | OM970165 + OM970225 | | Eastern Urals Reserve, Chelyabinsk region (30) | | Russia | | Urals | | 1 | | Original data | |
| AaUmt40 | KJ082026 + OM970227 | | Eastern Urals Reserve, Chelyabinsk region (30) | | Russia | | Urals | | 1 | | Original data | |
| AaUmt41 | OM970137 + OM970176 | | Aytuarskaya Steppe, Orenburg region (25) | | Russia | | Urals | | 4 | | Original data | |
| AaUmt42 | OM970138 + OM970177 | | Kinzebulatovo village, Baskortostan (27) | | Russia | | Urals | | 1 | | Original data | |
| AaUmt43 | OM970139 + OM970178 | | Dvurechensk village, Sverdlovsk region (34) | | Russia | | Urals | | 2 | | Original data | |
| AaUmt44 | OM970140 + OM970179 | | Dvurechensk village, Sverdlovsk region (34) | | Russia | | Urals | | 1 | | Original data | |
| AaUmt45 | OM970141 + OM970169 | | Belaya Kholunitsa, Kirov region (22) | | Russia | | Urals | | 1 | | Original data | |
| AaUmt46 | OM970141 + OM970180 | | Belaya Kholunitsa, Kirov region (22)  Chornaya Kholunitsa village, Kirov region (23) | | Russia | | Urals | | 1  1 | | Original data | |
| AaUmt47 | OM970141 + OM970181 | | Chornaya Kholunitsa village, Kirov region (23) | | Russia | | Urals | | 1 | | Original data | |
| AaUmt48 | OM970142 + OM970169 | | Starikova village, Sverdlovsk region (33) | | Russia | | Urals | | 1 | | Original data | |
| AaUmt49 | OM970131 + OM970182 | | Starikova village, Sverdlovsk region (33) | | Russia | | Urals | | 1 | | Original data | |
| AaUmt50 | OM970146 + OM970183 | | Mashkara village, Tyumen region (42) | | Russia | | Western Siberia | | 1 | | Original data | |
| AaUmt51 | OM970146 + OM970185 | | Mashkara village, Tyumen region (42) | | Russia | | Western Siberia | | 1 | | Original data | |
| AaUmt52 | KJ082026 + OM970187 | | Mashkara village, Tyumen region (42) | | Russia | | Western Siberia | | 1 | | Original data | |
| AaUmt53 | OM970129 + OM970177 | | Aytuarskaya Steppe, Orenburg region (25) | | Russia | | Urals | | 1 | | Original data | |
| AaUmt54 | OM970143 + OM970204 | | Nizhniy Tagil, Sverdlovsk region (40) | | Russia | | Urals | | 3 | | Original data | |
| AaUmt55 | OM970153 + OM970205 | | Nizhniy Tagil, Sverdlovsk region (40) | | Russia | | Urals | | 1 | | Original data | |
| AaUmt56 | OM970154 + OM970206 | | Nizhniy Tagil, Sverdlovsk region (40) | | Russia | | Urals | | 1 | | Original data | |
| AaUmt57 | OM970128 + OM970207 | | Nizhniy Tagil, Sverdlovsk region (40) | | Russia | | Urals | | 1 | | Original data | |
| AaUmt58 | OM970155 + OM970209 | | Ilmen Nature Reserve, Chelyabinsk region (31) | | Russia | | Urals | | 1 | | Original data | |
| AaUmt59 | OM970128 + OM970210 | | Nizhniy Tagil, Sverdlovsk region (40) | | Russia | | Urals | | 1 | | Original data | |
| AaUmt60 | OM970143 + OM970211 | | Nizhniy Tagil, Sverdlovsk region (40) | | Russia | | Urals | | 1 | | Original data | |
| AaUmt61 | OM970143 + OM970212 | | Nizhniy Tagil, Sverdlovsk region (40) | | Russia | | Urals | | 2 | | Original data | |
| AaUmt62 | OM970140 + OM970189 | | Sos'va village, Sverdlovsk region (39) | | Russia | | Urals | | 1 | | Original data | |
| AaUmt63 | OM970136 + OM970213 | | Sos'va village, Sverdlovsk region (39) | | Russia | | Urals | | 1 | | Original data | |
| AaAsmt05 | JN629047 | | - | | South Korea | | South Korea | | 1 | | Unpublished | |
| AaAsmt04 | HM034866 | | Jeju Island (79) | | South Korea | | Jeju Island | | 1 | | Unpublished) | |
| AsAsmt06 | HM034867 | | Mt. Jiri (59) | | South Korea | | South Korea | | 1 | | [22] | |
