# Supplementary material for: Phylogeography of the striped field mouse (Apodemus agrarius Pallas, 1771) in light of new data from central part of Northern Eurasia
Source: PLoS One. 2022 Oct 20;17(10):e0276466. doi: 10.1371/journal.pone.0276466 (PMC9584417; doi:10.1371/journal.pone.0276466)
Supplement: S2 Table — (DOC) [file pone.0276466.s002.doc]

**S2 Table. List of *cyt b* haplotypes, which together with haplotypes listed in S1 Table, used in variable sites distribution analysis**

| **GenBank assecion №** | **Length of *cyt* *b* fragment (bp)** | **References** |
| --- | --- | --- |
| AB096810 | 402 | [14] |
| AB096811 | 402 | [14] |
| AB096812 | 402 | [14] |
| AB096813 | 402 | [14] |
| AB096814 | 402 | [14] |
| AF264182 | 319 | Unpublished |
| AF264183 | 319 | Unpublished |
| AF264184 | 319 | Unpublished |
| AF264185 | 319 | Unpublished |
| AF264186 | 319 | Unpublished |
| AF264187 | 319 | Unpublished |
| AF264188 | 319 | Unpublished |
| AF427332 | 1081 | Unpublished |
| AF427333 | 1081 | Unpublished |
| AF427334 | 1081 | Unpublished |
| AJ311144 | 971 | Michaux et al., 2002 |
| AJ311145 | 971 | Michaux et al., 2002 |
| AM945740 | 1068 | [16] |
| AM945741 | 1059 | [16] |
| AM945742 | 1000 | [16] |
| AM945743 | 1067 | [16] |
| AM945744 | 1059 | [16] |
| AM945745 | 1060 | [16] |
| AM945746 | 1059 | [16] |
| AM945747 | 1009 | [16] |
| AM945748 | 1066 | [16] |
| AM945749 | 1068 | [16] |
| AM945750 | 1050 | [16] |
| AM945751 | 1068 | [16] |
| AM945752 | 1059 | [16] |
| AM945753 | 1055 | [16] |
| AM945755 | 1050 | [16] |
| AM945838 | 1078 | [16] |
| AM945839 | 1078 | [16] |
| AM945845 | 1078 | [16] |
| AM945846 | 1078 | [16] |
| AM945847 | 1078 | [16] |
| AM945848 | 1078 | [16] |
| AM945850 | 1063 | [16] |
| AY263609 | 264 at the 5' end; 340 at the 3' end | Doyle et al., 2003 |
| AY532796 | 392 | Unpublished |
| EU349733 | 1140 | Rowe et al., 2008 |
| GQ260167 | 435 | Schegel et al., 2009 |
| GQ260169 | 364 | Schegel et al., 2009 |
| GQ260170 | 364 | Schegel et al., 2009 |
| GQ260171 | 436 | Schegel et al., 2009 |
| GQ260172 | 258 | Schegel et al., 2009 |
| GQ260173 | 410 | Schegel et al., 2009 |
| GQ260174 | 600 | Schegel et al., 2009 |
| GQ260176 | 438 | Schegel et al., 2009 |
| GQ260177 | 258 | Schegel et al., 2009 |
| GQ260178 | 364 | Schegel et al., 2009 |
| GQ260179 | 387 | Schegel et al., 2009 |
| GQ260180 | 405 | Schegel et al., 2009 |
| GQ260181 | 407 | Schegel et al., 2009 |
| GQ260182 | 408 | Schegel et al., 2009 |
| GQ260183 | 437 | Schegel et al., 2009 |
| GQ260184 | 438 | Schegel et al., 2009 |
| GQ421162 | 258 | Schegel et al., 2009 |
| GQ421163 | 438 | Schegel et al., 2009 |
| GQ421164 | 413 | Schegel et al., 2009 |
| HQ343384 | 744 | Unpublished |
| HQ343385 | 744 | Unpublished |
| HQ343386 | 744 | Unpublished |
| HQ343387 | 744 | Unpublished |
| HQ343388 | 744 | Unpublished |
| HQ343389 | 744 | Unpublished |
| HQ343390 | 744 | Unpublished |
| HQ343391 | 744 | Unpublished |
| HQ343392 | 744 | Unpublished |
| HQ343393 | 744 | Unpublished |
| HQ343394 | 744 | Unpublished |
| HQ343395 | 744 | Unpublished |
| HQ343396 | 744 | Unpublished |
| HQ343397 | 744 | Unpublished |
| HQ343398 | 744 | Unpublished |
| HQ343399 | 744 | Unpublished |
| HQ343400 | 744 | Unpublished |
| HQ343401 | 744 | Unpublished |
| HQ343402 | 744 | Unpublished |
| HQ343403 | 744 | Unpublished |
| HQ343404 | 744 | Unpublished |
| HQ343405 | 744 | Unpublished |
| HQ343406 | 744 | Unpublished |
| JF318967 | 627 | Schegel et al., 2012 |
| KT278778 | 833 | Kim, Park, 2015 |
| KT278779 | 833 | Kim, Park, 2015 |
| KT278780 | 833 | Kim, Park, 2015 |
| KT278781 | 833 | Kim, Park, 2015 |
| KT278782 | 833 | Kim, Park, 2015 |
| KT278783 | 833 | Kim, Park, 2015 |
| KT278784 | 833 | Kim, Park, 2015 |
| KT278785 | 833 | Kim, Park, 2015 |
| KT278786 | 833 | Kim, Park, 2015 |
| KT278787 | 833 | Kim, Park, 2015 |
| KT278788 | 833 | Kim, Park, 2015 |
| KT278789 | 833 | Kim, Park, 2015 |
| KT278790 | 833 | Kim, Park, 2015 |
| KT278791 | 833 | Kim, Park, 2015 |
| KT278792 | 833 | Kim, Park, 2015 |
| KT278793 | 833 | Kim, Park, 2015 |
| KT278794 | 833 | Kim, Park, 2015 |
| KT278795 | 833 | Kim, Park, 2015 |
| KT278796 | 833 | Kim, Park, 2015 |
| KT278797 | 833 | Kim, Park, 2015 |
| KT278798 | 833 | Kim, Park, 2015 |
| KT278799 | 833 | Kim, Park, 2015 |
| KT278800 | 833 | Kim, Park, 2015 |
| KT278801 | 833 | Kim, Park, 2015 |
| KT278802 | 833 | Kim, Park, 2015 |
| KT278803 | 833 | Kim, Park, 2015 |
| KT278804 | 833 | Kim, Park, 2015 |
| KT278805 | 833 | Kim, Park, 2015 |
| KT278806 | 833 | Kim, Park, 2015 |
| KT278807 | 833 | Kim, Park, 2015 |
| KT278808 | 833 | Kim, Park, 2015 |
| KT278809 | 833 | Kim, Park, 2015 |
| KT278810 | 833 | Kim, Park, 2015 |
| KT278811 | 833 | Kim, Park, 2015 |
| KT278812 | 833 | Kim, Park, 2015 |
| KT278813 | 833 | Kim, Park, 2015 |
| KT278814 | 833 | Kim, Park, 2015 |
| KT278815 | 833 | Kim, Park, 2015 |
| KT278816 | 833 | Kim, Park, 2015 |
| KT278817 | 833 | Kim, Park, 2015 |
| KT278818 | 833 | Kim, Park, 2015 |
| KT278819 | 833 | Kim, Park, 2015 |
| KT278820 | 833 | Kim, Park, 2015 |
| KT278821 | 833 | Kim, Park, 2015 |
| KT278822 | 833 | Kim, Park, 2015 |
| KT278823 | 833 | Kim, Park, 2015 |
| KT278824 | 833 | Kim, Park, 2015 |
| KT278825 | 833 | Kim, Park, 2015 |
| KT278826 | 833 | Kim, Park, 2015 |
| KT278827 | 833 | Kim, Park, 2015 |
| KT278828 | 833 | Kim, Park, 2015 |
| KT278829 | 833 | Kim, Park, 2015 |
| KT278830 | 833 | Kim, Park, 2015 |
| KT278831 | 833 | Kim, Park, 2015 |
| KT279080 | 1092 | Unpublished |
| KT279081 | 1092 | Unpublished |
| KT279084 | 1092 | Unpublished |
| KT279089 | 1092 | Unpublished |
| KT279090 | 1092 | Unpublished |
| KT279091 | 1092 | Unpublished |
| KT279092 | 1092 | Unpublished |
| KT279093 | 1092 | Unpublished |
| KT279094 | 1092 | Unpublished |
| KT279099 | 1092 | Unpublished |
| KT279100 | 1092 | Unpublished |
| KT279101 | 1092 | Unpublished |
| KT279102 | 1092 | Unpublished |
| KT279103 | 1092 | Unpublished |
| KT279107 | 1092 | Unpublished |
| KT279108 | 1092 | Unpublished |
| KT279115 | 1092 | Unpublished |
| KT318765 | 897 | Unpublished |
| KT318766 | 897 | Unpublished |
| KT318775 | 897 | Unpublished |
| KT318777 | 897 | Unpublished |
| KT318784 | 897 | Unpublished |
| KX519422 | 897 | Unpublished |
| KX519424 | 897 | Unpublished |
| KX519425 | 897 | Unpublished |
| KX066073 | 1140 | Unpublished |
| KX756201 | 399 | Kim et al., 2018 |
| KX756202 | 399 | Kim et al., 2018 |
| KX756203 | 399 | Kim et al., 2018 |
| KX756204 | 399 | Kim et al., 2018 |
| KX756205 | 399 | Kim et al., 2018 |
| KX756219 | 374 | Kim et al., 2018 |
| KY753942 | 1140 | Steppan, Schenk, 2017 |
| MG748165 | 1071 | Liu et al., 2018 |
| MG748166 | 1071 | Liu et al., 2018 |
| MG748167 | 1071 | Liu et al., 2018 |
| MG748169 | 1071 | Liu et al., 2018 |
| MG748170 | 1071 | Liu et al., 2018 |
| MG748175 | 1071 | Liu et al., 2018 |
| MG748182 | 1071 | Liu et al., 2018 |
| MG748183 | 1071 | Liu et al., 2018 |
| MG748184 | 1071 | Liu et al., 2018 |
| MG748200 | 1071 | Liu et al., 2018 |
| MG748234 | 1071 | Liu et al., 2018 |
| MG748235 | 1071 | Liu et al., 2018 |
| MG748236 | 1071 | Liu et al., 2018 |
| MK329437 | 1046 | Ge et al., 2019 |
| MK329442 | 1046 | Ge et al., 2019 |
| MK329443 | 1046 | Ge et al., 2019 |
| MK329444 | 1046 | Ge et al., 2019 |
| MK329445 | 1046 | Ge et al., 2019 |
| MK329446 | 1046 | Ge et al., 2019 |
| MK329447 | 1046 | Ge et al., 2019 |
| MK329448 | 1046 | Ge et al., 2019 |
| MK329463 | 1046 | Ge et al., 2019 |
| MK329464 | 1046 | Ge et al., 2019 |
| MK329469 | 1046 | Ge et al., 2019 |
| MK329470 | 1046 | Ge et al., 2019 |
| MK329472 | 1046 | Ge et al., 2019 |
| MK329487 | 1046 | Ge et al., 2019 |
| MK329488 | 1046 | Ge et al., 2019 |
| MK329489 | 1046 | Ge et al., 2019 |
| MK329490 | 1046 | Ge et al., 2019 |
| MK329503 | 1046 | Ge et al., 2019 |
| MK329504 | 1046 | Ge et al., 2019 |
| MK329505 | 1046 | Ge et al., 2019 |
| MK329528 | 1046 | Ge et al., 2019 |
| MK329529 | 1046 | Ge et al., 2019 |
| MK329540 | 1046 | Ge et al., 2019 |
| MK329545 | 1046 | Ge et al., 2019 |
| MK329546 | 1046 | Ge et al., 2019 |
| MK329548 | 1046 | Ge et al., 2019 |
| MN122887 | 1140 | Unpublished |

**References for S2 Table**

Michaux JR, Chevert P, Filippucci M–G, Macholan M. Phylogeny of the genus *Apodemus* with a special emphasis on the subgenus *Sylvaemus* using the nuclear IRBP gene and two mitochondrial markers: cytochrome b and 12S rRNA. Mol Phylogenet Evol. 2002; 23: 123–136. doi: 10.1016/S1055-7903(02)00007-6 PMID: 12069545

Doyle CK, Davis BK, Cook RG, Rich RR, Rodgers JR. Hyperconservation of the N–formyl peptide binding site of M_3_: evidence that M_3_ is an old eutherian molecule with conserved recognition of a pathogen-associated molecular pattern. J Immunol. 2003; 171: 836–44. doi: 10.4049/jimmunol.171.2.836 PMID: 12847252

Rowe KC, Reno ML, Richmond DM, Adkins RM, Steppan SJ. Pliocene colonization and adaptive radiations in Australia and New Guinea (Sahul): multilocus systematics of the old endemic rodents (Muroidea: Murinae). Mol Phylogenet Evol. 2008; 47: 84–101. doi: 10.1016/j.ympev.2008.01.001 PMID: 18313945

Schlegel M, Klempa B, Auste B, Bemmann M, Schmidt–Chanasit J, Büchner Th et al. Dobrava-belgrade virus spillover infections, Germany. Emerg Infect Dis. 2009; 15: 2017–2020. doi: 10.3201/eid1512.090923 PMID: 19961690

Schlegel M, Ali HS, Stieger N, Groschup MH, Wolf R, Ulrich RG. Molecular identification of small mammal species using novel cytochrome *b* gene-derived degenerated primers. Biochem Genet. 2012; 50: 440–447. doi: 10.1007/s10528-011-9487-8 PMID: 22193288

Kim HR, Park YCh. Genetic diversity and genetic structure of the stripedfield mouse *Apodemus agrarius coreae* (Muridae,Rodentia) in Korea. Gene. 2015; 572: 292–297. doi: 10.1007/s42991-019-00001-0

Kim TW, Lee HJ, Kim YK, Oh HS, Han SH. Genetic identification of prey species from teeth in faeces from the Endangered leopard cat *Prionailurus bengalensis* using mitochondrial cytochrome b gene sequence. Mitochondrial DNA a DNA Mapp Seq Anal. 2018; 29: 170–174. doi: 10.1080/24701394.2016.1261852 PMID: 28093009

Steppan SJ, Schenk JJ. Muroid rodent phylogenetics: 900-species tree reveals increasing diversification rates. PLoS One. 2017; 12: e0183070. doi: 10.1371/journal.pone.0183070 PMID: 28813483

Liu SY, He K, Chen SD, Jin W, Murphy RW, Tang M-K, et al. How many species of *Apodemus* and *Rattus* occur in China? A survey based on mitochondrial cyt b and morphological analyses. Zool Res. 2018; 39: 309–320. doi: 10.24272/j.issn.2095-8137.2018.053 PMID: 29955026

Ge D, Feijo A, Cheng J, Lu L, Liu R, Abramov AV, et al. Evolutionary history of field mice (Murinae: Apodemus), with emphasis on morphological variation among species in China and description of a new species. Zool J Linn Soc. 2019; 187: 518–534. doi: 10.1093/zoolinnean/zlz032
