# Supplementary material for: Phylogeography of the striped field mouse (Apodemus agrarius Pallas, 1771) in light of new data from central part of Northern Eurasia
Source: PLoS One. 2022 Oct 20;17(10):e0276466. doi: 10.1371/journal.pone.0276466 (PMC9584417; doi:10.1371/journal.pone.0276466)
Supplement: S3 Table — Analysis was carried out using 593 complete and partial sequences, including both original and GenBank data (S1 and S2 Tables). (DOC) [file pone.0276466.s003.doc]

**S3 Table. The number (N) and percentage (%) of polymorphic sites of each fragment of *Apodemus agrarius* cytochrome *b* length 50 bp. Analysis was carried out using 593 complete and partial sequences, including both original and GenBank data (Table S1, Table S2).**

| ***сyt b* fragment (bp)** | **Polymorphic sites** | |
| --- | --- | --- |
|  | **N** | **%** |
| 1-50 | 12 | 3.85 |
| 51-100 | 17 | 5.45 |
| 101-150 | 14 | 4.49 |
| 151-200 | 11 | 3.53 |
| 201-250 | 12 | 3.85 |
| 251-300 | 14 | 4.49 |
| 301-350 | 14 | 4.49 |
| 351-400 | 13 | 4.17 |
| 401-450 | 10 | 3.21 |
| 451-500 | 17 | 5.45 |
| 501-550 | 8 | 2.56 |
| 551-600 | 15 | 4.81 |
| 601-650 | 12 | 3.85 |
| 651-700 | 12 | 3.85 |
| 701-750 | 19 | 6.09 |
| 751-800 | 18 | 5.77 |
| 801-850 | 12 | 3.85 |
| 851-900 | 16 | 5.13 |
| 901-950 | 14 | 4.49 |
| 951-1000 | 19 | 6.09 |
| 1001-1050 | 13 | 4.17 |
| 1051-1100 | 12 | 3.85 |
| 1101-1140 | 8 | 2.56 |
|  | ∑ = 312 | Median = 4.17 |
