# Supplementary figures and images for: Phylogeography of the striped field mouse (Apodemus agrarius Pallas, 1771) in light of new data from central part of Northern Eurasia
Source: PLoS One. 2022 Oct 20;17(10):e0276466. doi: 10.1371/journal.pone.0276466 (PMC9584417; doi:10.1371/journal.pone.0276466)

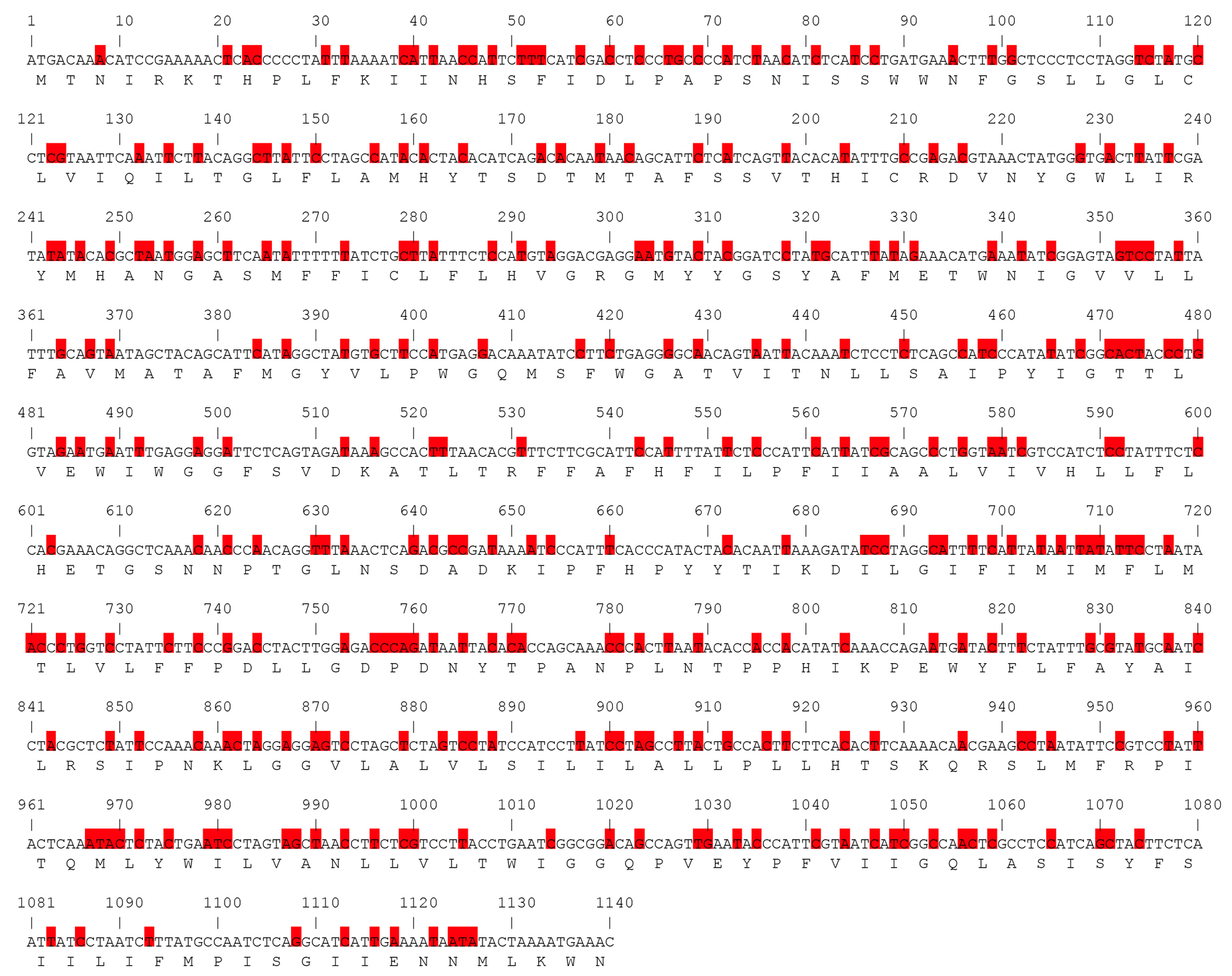

Supplement: S1 Fig — (TIF) [file pone.0276466.s004.tif]
